# Interaction with PALB2 Is Essential for Maintenance of Genomic Integrity by BRCA2

**DOI:** 10.1371/journal.pgen.1006236

**Published:** 2016-08-04

**Authors:** Suzanne A. Hartford, Rajanikant Chittela, Xia Ding, Aradhana Vyas, Betty Martin, Sandra Burkett, Diana C. Haines, Eileen Southon, Lino Tessarollo, Shyam K. Sharan

**Affiliations:** 1 Mouse Cancer Genetics Program, Center for Cancer Research, National Cancer Institute, Frederick, Maryland, United States of America; 2 Leidos Biomedical Inc., National Cancer Institute, Frederick, Maryland, United States of America; St Jude Children's Research Hospital, UNITED STATES

## Abstract

Human breast cancer susceptibility gene, *BRCA2*, encodes a 3418-amino acid protein that is essential for maintaining genomic integrity. Among the proteins that physically interact with BRCA2, Partner and Localizer of BRCA2 (PALB2), which binds to the N-terminal region of BRCA2, is vital for its function by facilitating its subnuclear localization. A functional redundancy has been reported between this N-terminal PALB2-binding domain and the C-terminal DNA-binding domain of BRCA2, which undermines the relevance of the interaction between these two proteins. Here, we describe a genetic approach to examine the functional significance of the interaction between BRCA2 and PALB2 by generating a knock-in mouse model of *Brca2* carrying a single amino acid change (Gly25Arg, *Brca2*^*G25R*^) that disrupts this interaction. In addition, we have combined *Brca2*^*G25R*^ homozygosity as well as hemizygosity with *Palb2* and *Trp53* heterozygosity to generate an array of genotypically and phenotypically distinct mouse models. Our findings reveal defects in body size, fertility, meiotic progression, and genome stability, as well as increased tumor susceptibility in these mice. The severity of the phenotype increased with a decrease in the interaction between BRCA2 and PALB2, highlighting the significance of this interaction. In addition, our findings also demonstrate that hypomorphic mutations such as *Brca2*^*G25R*^ have the potential to be more detrimental than the functionally null alleles by increasing genomic instability to a level that induces tumorigenesis, rather than apoptosis.

## Author Summary

Inheritance of a deleterious mutation in *Breast Cancer2* (*BRCA2*) is a well-established factor associated with increased risk of hereditary breast and ovarian cancers. BRCA2 has numerous roles in maintaining the genome to prevent accumulation of mutations that can lead to cancer formation. Here, we describe the generation of a novel mouse model that has a single amino acid alteration in the BRCA2 protein that affects its interaction with another hereditary breast cancer protein, PALB2. We use these mice to examine the relevance of the interaction between BRCA2 and PALB2. We also examine the effect of disrupting one copy of *Palb2* gene in the mutant mice. Our findings show that the loss of interaction between the two proteins increases cancer formation in mice. We conclude that not only are BRCA2 and PALB2 required for tumor suppression, their physical interaction is equally important.

## Introduction

Error free DNA repair and replication along with proper cell cycle checkpoints are essential for maintaining the stability of the genome. The breast cancer susceptibility gene, *BRCA2*, is a major player involved in these functions. BRCA2 recruits the DNA recombinase, RAD51, to the site of double strand breaks (DSBs), which mediates repair by homologous recombination (HR) [1–3]. During replication stress, BRCA2 protects the nascent DNA strand at the stalled replication forks to prevent the forks from collapsing allowing their recovery [4–6]. BRCA2 is involved in the intra-S-phase as well as the G2/M checkpoints that prevent premature mitotic entry of cells with damaged DNA [7–9]. These critical functions of BRCA2 are key to its ability to maintain the genomic integrity and contribute to its role as a tumor suppressor. Although BRCA2 mutation carriers predominantly have an increased risk of hereditary breast and ovarian cancer (HBOC), they are also at risk of other cancers such as pancreatic, gastric, laryngeal and prostate cancers [10]. Biallelic mutations in *BRCA2* are associated with Fanconi anemia (FA), a rare autosomal recessive disorder characterized by sensitivity to DNA crosslinking agents, bone marrow failure, developmental abnormalities, and increased tumorigenesis [11].

**Fig 1:**
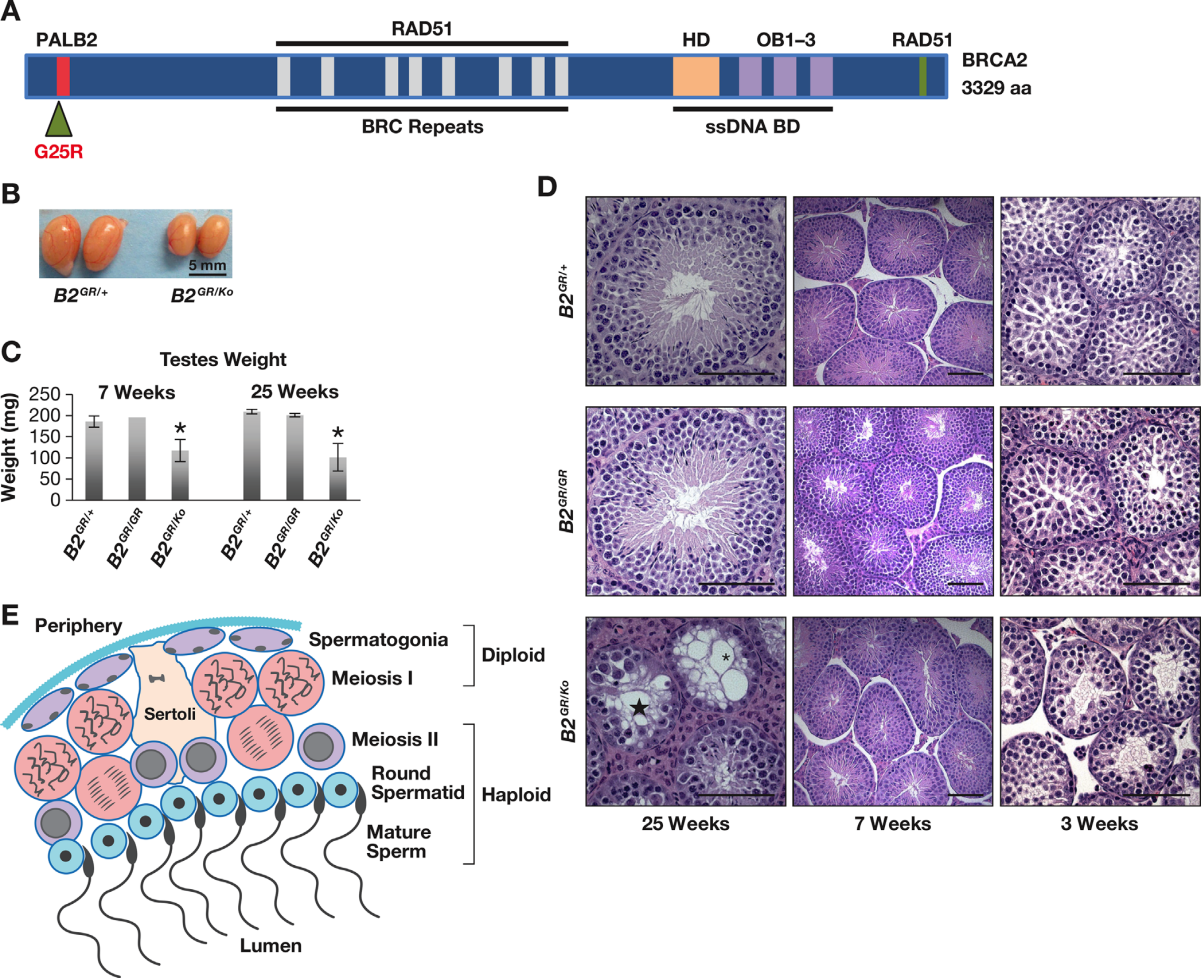
*Brca2*^*G25R/Ko*^ mutant males have decreased fertility. **A.** Structural and functional domains of murine BRCA2 consisting of 3329 amino acids with the N-terminal PALB2 binding domain (red), BRC repeats (gray) in the middle, helical domain (HD, orange), oligonucleotide binding (OB) regions 1–3 (purple), and a C-terminal RAD51 binding domain (green). Single-strand DNA binding domain (ssDNA, line). The dark green triangle marks the location of the G25R mutation. **B.** Representative images of mouse testes of indicated genotype (*B2*^*GR/+*^, left and *B2*^*GR/Ko*^, right) at 25 weeks of age. **C.** Average paired testes weight (mg) of the listed genotypes at 7 weeks and 25 weeks, error bars indicate SD. * p<0.05. **D.** Representative H&E stained histology of testes of the indicated genotypes (*B2*^*GR/+*^, upper, *B2*^*GR/GR*^, middle, and *B2*^*GR/Ko*^, lower) at 25 weeks (tubules lacking germ cells (*), and tubules arrest at meiosis (star), 7 weeks with normal spermatogenesis, and 3 weeks, with reduced haploid cells in *B2*^*GR/Ko*^ testis sections. **E.** Cartoon of a portion of mouse adult seminiferous tubule cross-section showing spermatozoa at various developmental stages from periphery (top) to lumen (bottom). Scale Bar = 100 μM. Controls are represented by either: *Brca2*^*+/+*^, *Brca2*^*Ko/+*^, or *Brca2*^*G25R/+*^. Abbreviations: *Brca2*^*G25R/+*^ = ***B2***^***GR/+***^, *Brca2*^*G25R/G25R*^ = ***B2***^***GR/GR***^, *Brca2*^*G25R/Ko*^ = ***B2***^***GR/Ko***^.

BRCA2 executes its basic functions through its interaction with a number of different proteins, of which bind to the various functional domains of BRCA2 (Fig 1A) [10, 12]. One of these proteins is Partner and Localizer of BRCA2 (PALB2), which binds to the N-terminal region of BRCA2 and is necessary for proper sub-nuclear localization of BRCA2 [13]. *In vitro* studies have suggested PALB2 to be essential for many functions of BRCA2, such as G2/M checkpoint, replication fork protections, as well as repair of double strand breaks by HR [7, 14–16].

Mutations in *PALB2* are associated with increased risk of breast cancer, similar to that of *BRCA2*, making it an important hereditary breast cancer susceptibility gene [17]. Like *BRCA2*, *PALB2* is also classified as an FA gene (FANCN). With so many similar and overlapping functions, it is not surprising that the *Palb2* null mice exhibit an early embryonic lethal phenotype similar to *Brca2* null embryos. K14-Cre-mediated deletion of *Palb2* loss results in mammary tumors albeit with long latency, which is significantly reduced when combined with *Trp53* loss. Interestingly, the genomic profiles of *Palb2;Trp53*-loss associated tumors are different from those associated with *Brca2;Trp53*-deficient tumors in mice [18–20].

The domains of BRCA2 and PALB2 interaction have been well characterized. In BRCA2, the highly conserved 10–40 residues near the N-terminus were shown to be necessary and sufficient for binding to PALB2. Within this region, three cancer-associated mutations affecting two amino acids, namely Gly25 (changed to Arg, G25R) and Trp31 (changed to Cys or Arg, W31C and W31R, respectively) were found to disrupt the interaction with PALB2. Furthermore, while W31C and W31R completely abolished the interaction with PALB2, G25R significantly reduced but did not completely abolish the interaction [13]. A mouse embryonic stem (mES) cell-based functional assay was used to examine the effect of these three mutations on BRCA2 function and results were consistent with biochemical studies [21]. The W31C and W31R mutations affected viability of mES cells. In contrast, the G25R mutation resulted in viable mES cells that exhibited sensitivity to DNA damaging agents and a significant reduction in homologous recombination. These results suggested that although G25R disrupted the interaction of BRCA2 with PALB2, the residual interaction was sufficient for cell viability, but not for DNA repair function [21].

Functional characterization of cancer-causing mutations in *PALB2* suggested that the C-terminal region containing four WD40 motifs binds to BRCA2 [22]. It was subsequently shown that WD40 repeats 3 and 4 are required for this binding. X-ray crystallography of BRCA2 peptide (residues 21–39) and PALB2 (C-terminal region, residues 836–1186) co-crystals revealed that the core of the interaction is provided by Trp 31, Phe 32 and Leu 35 residues of BRCA2 [22, 23]. This explained the severity of the phenotype observed in mES cells expressing W31C and W31R mutant BRCA2. Interestingly, the N-terminal region of PALB2 binds to BRCA1, a breast cancer associated protein involved in DNA damage response and repair, and this interaction is impaired in G1 through the ubiquitination of PALB2 [16, 24]. PALB2 is believed to be a molecular adaptor that functionally link BRCA1 and BRCA2, which is apparently essential for BRCA2-mediated RAD51 recruitment and HR [16]. PALB2 also binds RAD51 in the same region as it binds BRCA2 [23, 25, 26].

While the binding of PALB2 to BRCA2 is considered vital for BRCA2 function, recent studies have suggested that there may exist a functional redundancy between the PALB2-binding domain and the C-terminal DNA-binding domain of BRCA2. In chicken DT40 cells, when the PALB2-binding N-terminal region of BRCA2 was deleted, it resulted in a mild reduction in HR and a mild sensitivity to IR, camptothecin, cisplatin as well as olaparib, a PARP1 inhibitor [14]. These cells proliferated with nearly normal kinetics and had a mild increase in the number of spontaneous chromosomal aberrations. In contrast, the loss of the BRCA2 C-terminal DBD, resulted in a moderate sensitivity to these DNA damaging agents as well as an increase in chromosomal aberrations and reduction in HR, suggesting that the N-terminal PALB2 binding region is dispensable but the DBD is necessary for DNA repair. Interestingly, when both regions were deleted, the effects were compounded resulting in a phenotype very similar to *Brca2-null* DT40 cells. These findings suggest that the two regions may be playing significantly overlapping roles [14]. Another study using BRCA2 polypeptides of various lengths revealed that having either the PALB2 binding region or the DBD is sufficient for BRCA2–mediated homologous recombination [15]. Taken together, these observations have raised concerns over the significance of the interaction between BRCA2 and PALB2, especially on the tumor suppressor function of BRCA2.

To examine the functional consequences of disrupting the interaction between BRCA2 and PALB2 and ascertain the requirement of this interaction for BRCA2 function *in vivo*, we have generated a knock-in mouse model, with a point mutation (c.73G>A) in exon 3 of *Brca2* that changes the highly conserved glycine at position 25 to arginine (S1 Fig and Fig 1A). Although mice carrying the *Brca2*^*G25R*^ mutant allele in a heterozygous or homozygous state exhibited no overt phenotype, we observed reduced fertility and a significant increase in tumor predisposition in hemizygous mutant mice. These phenotypes are significantly exacerbated when combined with *Palb2* heterozygosity. Functional characterization of these mice unequivocally demonstrates the importance of BRCA2-PALB2 interaction on BRCA2-mediated genome stability as well as tumor suppression.

## Results

### *Brca2*^*G25R/G25R*^ mice are viable

To examine the effect of disrupted interaction between BRCA2 and PALB2, we generated *Brca2*^*G25R/+*^ mice by introducing a G>A substitution in the first base of codon 25 of *Brca2* in exon 3 in mouse embryonic stem (ES) cells by gene targeting (S1 Fig, Fig 1A). These mice are viable and fertile. When *Brca2*^*G25R/+*^ mice were intercrossed, homozygous mutants were obtained at expected Mendelian ratios (Table 1, p = 0.97). The *Brca2*^*G25R/G25R*^ mice have a normal body size, both males and females are fertile, and have no observable phenotype.

**Table 1:** Observed and expected birth ratio and Chi Square analysis of offspring of various genotypes from *Brca2*^*GR/+*^ intercross.

	*Brca2*^*+/+*^	*Brca2*^*GR/+*^	*Brca2*^*GR/GR*^
Observed	54	106	55
Expected	54	108	54

χ^2^ p-value 0.97

We next examined the phenotype associated with the *Brca2*^*G25R*^ allele in a hemizygous state, which mimics the physiological state of cells undergoing loss of heterozygosity in *BRCA2* mutation carriers. We crossed *Brca2*^*G25R/G25R*^ with mice heterozygous (*Brca2*^*Ko/+*^) for a *Brca2* null allele, to obtain *Brca2*^*G25R/Ko*^ mice. These mice are also viable and born at expected Mendelian ratios (Table 2, p = 0.90). Female mice are fertile with no histological or developmental defect, yet the male mice exhibit a decline in their fertility by 20–25 weeks of age. To determine the cause of the decline in fertility, we examined the testes of *Brca2*^*G25R/Ko*^ males. At 25 and 7 weeks of age, we found the testes to be significantly reduced in size relative to the control littermates (48% and 60% respectively of control (wildtype and *Brca2*^*Ko/+*^ mice), Fig 1B and 1C, p = 0.01 and p<0.01). In addition, there was a 40% reduction in the sperm count. Histological analysis of the testis of *Brca2*^*G25R/Ko*^ males at 25 weeks showed the presence of some seminiferous tubules that were filled with spermatocytes and appeared similar to the tubules present in *Brca2*^*G25R/+*^ and *Brca2*^*G25R/G25R*^ males (Fig 1D, left panel). However, they also had some seminiferous tubules that were either completely devoid of germ cells (Fig 1D, asterisks) or with spermatocytes in meiotic arrest (Fig 1D, star, and Fig 1E). Histologically, the testis at 7 weeks appeared normal and indistinguishable from the testis of *Brca2*^*G25R/+*^ and *Brca2*^*G25R/G25R*^ littermates (Fig 1D center panel), with the seminiferous tubules filled with spermatocytes at different developmental stages (Fig 1E).

**Table 2:** Observed and expected birth ratio and Chi Square analysis of offspring of various genotypes from *Brca2*^*GR/GR*^ X *Brca2*^*Ko/+*^ cross.

	*Brca2*^*GR/+*^	*Brca2*^*GR/Ko*^
Observed	30	29
Expected	30	30

χ^2^ p-value 0.90

To examine the testis morphology when the first wave of meiosis starts, we examined 3 week-old males (prior to completion of spermatogenesis). Histological analysis of *Brca2*^*G25R/Ko*^ testis revealed the presence of more of the larger meiotic cells and less of the smaller haploid cells in the lumen when compared to their control littermates, suggesting a delay or defect in the cells to progress through meiosis (Fig 1D right panels, and Fig 1E).

### Delayed meiotic progression due to persistent DNA damage

**Fig 2:**
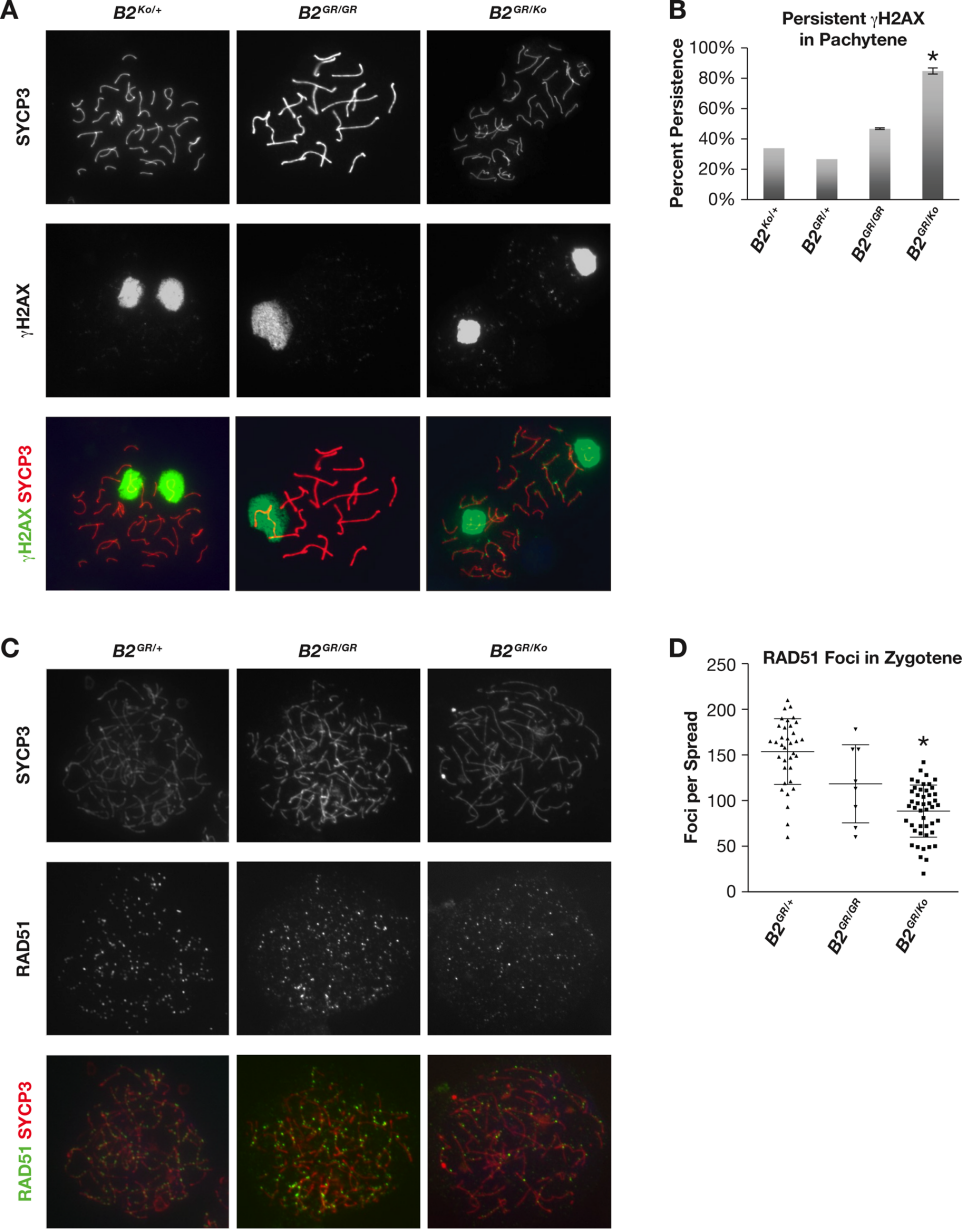
*Brca2*^*G25R/Ko*^ decreased testis size due to persistent DSBs and decreased localization of RAD51 in meiocytes. **A.** Representative images of pachytene meiocytes of (*B2*^*Ko/+*^, left, *B2*^*GR/GR*^, center, and *B2*^*GR/Ko*^, right) stained with SYCP3 (top, red), γH2AX (middle, green), and merged (bottom). **B.** Quantification of cells containing persistent γH2AX staining in pachynema. **C.** Representative images of zygotene meiocytes of (*B2*^*GR/+*^, left, *B2*^*GR/GR*^, center, and *B2*^*GR/Ko*^, right) stained with SYCP3 (top, red), RAD51 (middle, green), and merged (bottom). **D.** Quantification of RAD51 foci along SYCP3 stained meiotic cores at zygonema. Error bars: SD, * p<0.05. Controls are represented by either: *Brca2*^*+/+*^, *Brca2*^*Ko/+*^, or *Brca2*^*G25R/+*^. Abbreviations: *Brca2*^*Ko/+*^ = ***B2***^***Ko/+***^, *Brca2*^*G25R/+*^ = ***B2***^***GR/+***^, *Brca2*^*G25R/G25R*^ = ***B2***^***GR/GR***^, *Brca2*^*G25R/Ko*^ = ***B2***^***GR/Ko***^.

To determine the precise defect in meiotic progression, we examined meiocytes in pachynema stage of prophase I by staining with SYCP3, a marker of the synaptonemal complex, and γH2AX, a mark of unrepaired DNA double strand breaks (DSBs) [27]. As seen in *Brca2*^*Ko/+*^ and *Brca2*^*G25R/G25R*^ chromosome spreads (Fig 2A), by this meiotic stage most DSBs are repaired and γH2AX staining is restricted to the unsynapsed sex chromosomes. In contrast, *Brca2*^*G25R/Ko*^ meiocytes revealed a persistent staining of γH2AX (66% of spreads, p = 0.04) along the synapsed chromosomes suggesting the presence of unrepaired DSB breaks (Fig 2A and 2B). We also examined the presence of RAD51 foci at pachynema, which is restricted to 0–2 visible foci per pair of synapsed chromosomes and multiple RAD51 foci or a cloud along the sex chromosomes. In the *Brca2*^*G25R/Ko*^ meiocytes the number of foci along the sex chromosomes is significantly reduced when compared to littermates (S2 Fig (arrows), p<0.001). Similarly, at zygonema, an earlier stage of meiotic prophase I, we observed an average of only 88 RAD51 foci per *Brca2*^*G25R/Ko*^ meiocytes, which is significantly reduced compared to the controls at 154 foci (Fig 2C and 2D, p<0.001). These observations show that a decrease in BRCA2-PALB2 interaction affects RAD51 recruitment to the sites of DSBs during meiosis.

### Reduced *Brca2*^*G25R/Ko*^ testis size due to increased apoptosis

With the delay of progression through meiosis and the persistence of DNA damage in meiotic spreads, we examined if this leads to increased apoptosis, which would account for the overall decrease in testis size. We performed TUNEL staining on 3-week old mouse testis sections but observed no significant difference in the number of TUNEL positive cells per tubule between different genotypes (S3A and S3C Fig). We then examined the testis of adult animals to see if the delay in meiotic progression may result in cells with unrepaired DNA were then cleared at a later time-point. Indeed, in 7-week old mice, we detected a significant increase in the number of TUNEL positive cells in the *Brca2*^*G25R/KO*^ males (S3B and S3D Fig, p = 0.01) compared to littermate controls. These findings suggest that the declining fertility may be due to persistent DNA damage that leads to apoptosis of the spermatogonia, causing a gradual change in the composition of the seminiferous tubule.

### *Brca2*^*G25R*^ animals have increased tumor susceptibility

**Fig 3:**
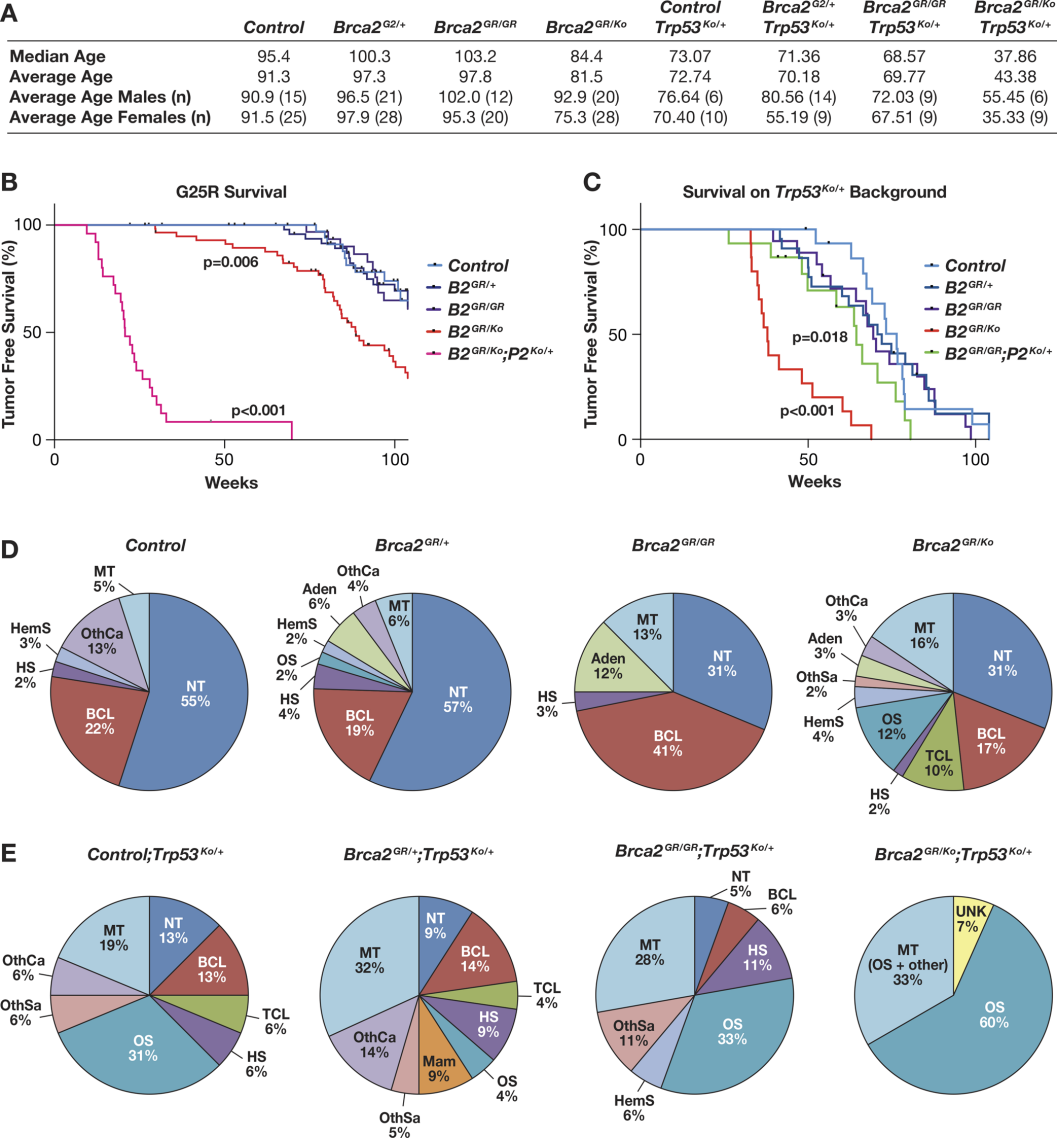
Increased tumor predisposition in *Brca2*^*G25R*^ hemizygous mice. **A.** Median and average age of death, including number of mice per genotype analyzed (n) and shown by gender. **B.** Kaplan-Meier tumor-free survival curves of *Brca2* mutant animals (104 week study). **C.** Kaplan-Meier tumor-free survival curves of *Brca2* mutant mice on a *Trp53*^*Ko/+*^ genetic background (104 week study). **D.** and **E.** Distribution of tumor types found in mice of indicated genotypes. Abbreviations: NT = No tumor observed, BCL = B-cell lymphoma, TCL = T-cell lymphoma, HS = Histiocytic sarcoma, OS = Osteosarcoma, HemS = Hemangiosarcoma, OthSa = Other sarcomas, AD = Adenomas, OthCa = Other carcinomas, MT = Multiple tumors, UNK = cause of death unknown. * UNK are included in the tumor-free survival curves as potential neoplasias. Controls are represented by: Brca2^+/+^ and Brca2^Ko/+^. Abbreviations: Brca2^Ko/+^ = **B2**^**Ko/+**^, Brca2^G25R/+^ = **B2**^**GR/+**^, Brca2^G25R/G25R^ = **B2**^**GR/GR**^, Brca2^G25R/Ko^ = **B2**^**GR/Ko**^.

To examine the relevance of physical interaction between BRCA2 and PALB2 on the tumor suppressor function of BRCA2, we generated a cohort of control (wild-type and *Brca2*^*Ko/+*^), *Brca2*^*G25R/+*^, *Brca2*^*G25R/G25R*^, and *Brca2*^*G25R/Ko*^ animals and monitored their tumor free survival for 2 yrs. The mean survival of the wild-type and *Brca2*^*Ko/+*^ mice (91 weeks) as well as *Brca2*^*G25R/+*^ mice (97 weeks) was not significantly different from the mean survival of *Brca2*^*G25R/G25R*^ and *Brca2*^*G25R/Ko*^ animals (98, 81 weeks, respectively, Fig 3A). Interestingly there is a difference in tumor latency of the *Brca2*^*G25R/Ko*^ when broken down by gender. The mean survival of females is 75 weeks, whereas the mean survival of males is 93 weeks (Fig 3A). Based on a Kaplan-Meier curve, the tumor-free survival of *Brca2*^*G25R/Ko*^ was significantly different from the control animals (Fig 3B, red line, Gehan-Breslow-Wilcoxcon p = 0.006). In the wild-type and *Brca2*^*Ko/+*^ mice, 55% of the mice did not exhibit any signs of neoplasia, however 22% of mice died due to B-cell lymphomas, with the remaining mice exhibiting various other tumor types, and 5% of these had multiple tumors (Fig 3D, S4 Fig). The cause of death and tumor spectrum of *Brca2*^*G25R/+*^ mice was similar to the controls (Fig 3D, S4 Fig) In contrast, 41% of the *Brca2*^*G25R/G25R*^ animals died due to B-cell lymphomas, another 28% had various other tumor types, with only 31% of the mice not exhibiting any signs of neoplasia (Fig 3D, S4 Fig). The tumor spectrum of *Brca2*^*G25R/Ko*^ mice was remarkably different from the other groups. While 17% of the mice developed B-cell lymphomas, an additional 10% of mice developed T-cell lymphomas, 42% with other tumor types and 16% of these had multiple tumors. However, 31% of these animals did not exhibit any signs of neoplasias (Fig 3D, S4 Fig,). This increased tumor predisposition of *Brca2*^*G25R/KO*^ mice suggests that the physical interaction of BRCA2 with PALB2 is critical for their tumor suppressor function.

### *Brca2*^*G25R*^ hemizygous mice have a propensity towards osteosarcomas on a *Trp53* heterozygous genetic background

It has been previously reported that the tumor susceptibility of *Brca2* mutant mice is enhanced on a *Trp53* mutant genetic background [28]. Therefore, we generated *Brca2*^*G25R*^ mutant mice of various genotypes on a *Trp53*^*Ko/+*^ genetic background. The mean survival of *Trp53*^*Ko/+*^ mice was 73 weeks (Fig 3A). Osteosarcoma was detected in 31% of the mice, 19% developed lymphomas (B- and T-cell), 19% of the animals had multiple tumors, with only 13% showing no signs of neoplasias (Fig 3C, Fig 3E, S5 Fig). In this cohort, we observed a significant reduction in the mean survival (p<0.001) of *Brca2*^*G25R/Ko*^*;Trp53*^*Ko/+*^ mice to 43 weeks and a high incidence of tumor formation compared to mice of other genotypic classes (Fig 3C, Fig 3E, S5 Fig). Almost all *Brca2*^*G25R/Ko*^*;Trp53*^*Ko/+*^ mice succumbed to osteosarcomas (93% of the animals). Of these, a third also had additional tumors types (Fig 3E, S5 Fig). The *Brca2*^*G25R/+*^*;Trp53*^*Ko/+*^ mice had a mean survival of 70 weeks. 18% of the mice developed lymphomas (B- and T-cell), 37% had other carcinomas, 32% had multiple tumors, and only 4% developed osteosarcomas (Fig 3E, S5 Fig). While the *Brca2*^*G25R/G25R*^*;Trp53*^*Ko/+*^ mice had a comparable mean survival (70 weeks), 33% developed osteosarcomas. In addition, 17% had other carcinomas, with 22% of the animals having multiple tumors and 11% developed B-cell lymphomas (Fig 3E, S5 Fig). The difference in tumor latency based on gender is also evident in these mice; *Brca2*^*G25R/+*^*;Trp53*^*Ko/+*^ females exhibited an average latency of 55 weeks, yet it was 81 weeks for males. Similarly, *Brca2*^*G25R/Ko*^*;Trp53*^*Ko/+*^ males exhibited an average tumor latency of 55 weeks but the average female tumor latency was 35 weeks. Taken together, these data show a significant reduction in the tumor latency and a significant change in the tumor spectrum of *Brca2*^*G25R*^ hemizygous mice on a *Trp53* heterozygous genetic background that is also influenced by gender.

### Synergistic interaction between *Brca2*^*G25R*^ and *Palb2* heterozygosity

**Fig 4:**
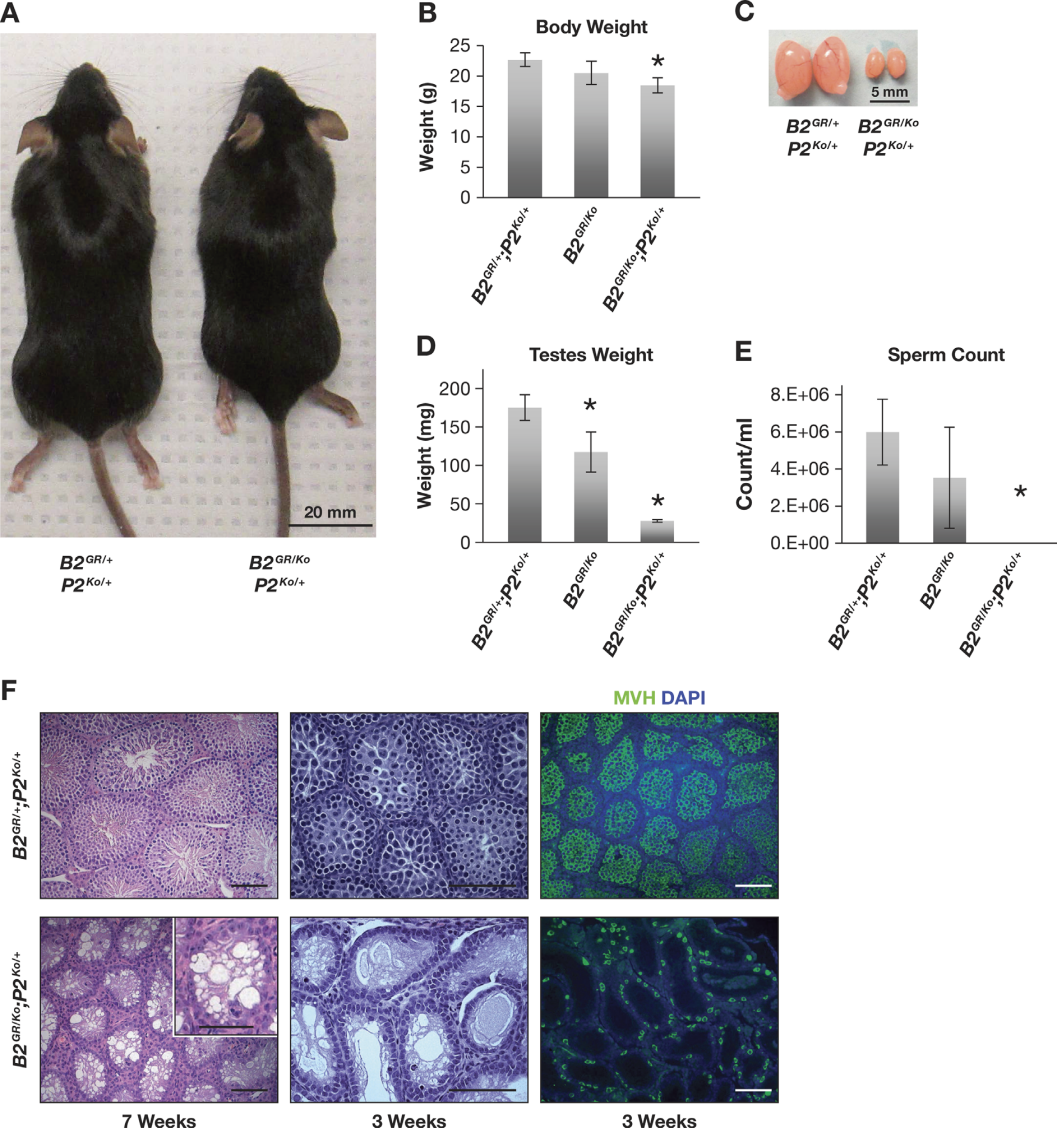
Synergistic interaction between *Brca2*^*G25R*^ mutation and *Palb2* heterozygosity. **A.** Image of decreased body size in *B2*^*GR/Ko*^*;P2*^*Ko/+*^ animals (right) compared to littermate controls (left). **B.** Average body weight of mice of indicated genotypes at 7 weeks. **C.** Seven-week old testes of *B2*^*GR/Ko*^*;P2*^*Ko/+*^ animals (right) are smaller compared to littermate controls (left). **D.** Average weight of testes of indicated genotypes at 7 weeks. **E.** Average sperm count of mice of indicated genotypes at 7 weeks. **F.** Representative images of testes sections from *B2*^*GR/+*^*;P2*^*Ko/+*^ (upper) and *B2*^*GR/Ko*^*;P2*^*Ko/+*^ (lower) animals. Left panel: 7 week H&E images, notice loss of germ cells in seminiferous tubules. Middle Panel: 3 week H&E stained images. Right panel: 3 week testis sections stained with MVH (green) and DAPI (blue) to visualize the germ cells. Controls are represented by either: *Brca2*^*+/+*^, *Brca2*^*Ko/+*^, or *Brca2*^*G25R/+*^ (with or without *Palb2*^*Ko/+*^). Abbreviations: *Brca2*^*G25R/+*^ = ***B2***^***GR/+***^, *Brca2*^*G25R/Ko*^ = ***B2***^***GR/Ko***^, *Palb2*^*Ko/+*^ = ***P2***^***Ko/+***^.

To fully appreciate whether the phenotypes observed in *Brca2*^*G25R/G25R*^ as well as *Brca2*^*G25R/Ko*^ mice are due to decreased interaction of BRCA2 with PALB2, we examined the effect of the loss of one functional allele of *Palb2*. We crossed *Brca2*^*G25R/Ko*^ mice to *Palb2* heterozygous mice carrying a gene trap allele known to render the gene non-functional. *Palb2*^*Ko/+*^ mice are viable and fertile with no known deleterious phenotype including tumor susceptibility. *Palb2*^*Ko/Ko*^ mice are not viable and die during early embryogenesis [18–20]. Both *Brca2*^*G25R/G25R*^*;Palb2*^*Ko/+*^ (p = 0.24, Table 3) as well as *Brca2*^*G25R/Ko*^*;Palb2*^*Ko/+*^ mice are viable and born at expected Mendelian ratios (p = 0.48, Table 4). The *Brca2*^*G25R/G25R*^*;Palb2*^*Ko/+*^ mice are phenotypically indistinguishable from their control littermates. However, *Brca2*^*G25R/Ko*^*;Palb2*^*Ko/+*^ mice have significantly reduced body size compared to their normal littermates (19% reduction, p = 0.003, Fig 4A and 4B). In addition, the *Brca2*^*G25R/Ko*^*;Palb2*^*Ko/+*^ males are infertile with a significant reduction in testis size compared to the control at 6–8 weeks (14% of control, Fig 4C and 4D). No sperm was detected in these animals, suggesting a severe defect in spermatogenesis (Fig 4E).

**Table 3:** Observed and expected birth ratio and Chi Square analysis of offspring of various genotypes from *Brca2*^*GR/+*^*;Palb2*^*ko/+*^ X *Brca2*^*GR/+*^ cross.

	*Brca2*^*+/+*^		*Brca2*^*GR/+*^		*Brca2*^*GR/GR*^	
	*Palb2*^*+/+*^	*Palb2*^*Ko/+*^	*Palb2*^*+/+*^	*Palb2*^*Ko/+*^	*Palb2*^*+/+*^	*Palb2*^*Ko/+*^
Observed	12	9	27	15	13	17
Expected	12	12	24	24	12	12

χ^2^ p-value = 0.24

**Table 4:** Observed and expected birth ratio and Chi Square analysis of offspring of various genotypes from *Brca2*^*GR/+*^*;Palb2*^*ko/+*^ X *Brca2*^*Ko/+*^ cross.

	*Brca2*^*+/+*^		*Brca2*^*Ko/+*^		*Brca2*^*GR/+*^		*Brca2*^*GR/Ko*^	
	*Palb2*^*+/+*^	*Palb2*^*Ko/+*^	*Palb2*^*+/+*^	*Palb2*^*Ko/+*^	*Palb2*^*+/+*^	*Palb2*^*Ko/+*^	*Palb2*^*+/+*^	*Palb2*^*Ko/+*^
Observed	21	19	18	18	14	13	16	9
Expected	16	16	16	16	16	16	16	16

χ^2^ p-value = 0.48

Histological analysis of the testes of *Brca2*^*G25R/Ko*^*;Palb2*^*Ko/+*^ mice at 6–8 weeks of age revealed that the seminiferous tubules only had sertoli cells and were completely devoid of germ cells (Fig 4F, left panel), while the *Brca2*^*G25R/+*^*;Palb2*^*Ko/+*^, *Brca2*^*Ko/+*^*;Palb2*^*Ko/+*^, and *Brca2*^*GR/GR*^*;Palb2*^*Ko/+*^ males proceeded through spermatogenesis normally (Fig 4F and S6A Fig). The lack of any defect in *Brca2*^*Ko/+*^*;Palb2*^*Ko/+*^ mice suggests that the mutant phenotype observed in *Brca2*^*G25R/Ko*^*;Palb2*^*Ko/+*^ mice is likely due to the *Brca2*^*G25R*^ mutation and not due to the reduced levels of both BRCA2 and PALB2 proteins. To determine whether there was a complete failure of germ cell formation, we examined the testes of mice at 3 weeks of age during initiation of meiosis. This was the age when we first observed defects in the *Brca2*^*G25R/Ko*^ mice. In the testis section of 3-week old *Brca2*^*G25R/Ko*^*;Palb2*^*Ko/+*^ mice, in addition to sertoli cells along the basal lamina, we observed cells that appeared to be germ cells (Fig 4F, center panel, S6A Fig, Fig 1E). To confirm the identity of these cells, we stained the testis sections with a germ cell marker, mouse Vasa homologue (MVH) [29]. We observed several MVH-positive cells, which shows that the *Brca2*^*G25R/Ko*^*;Palb2*^*Ko/+*^ males are not devoid of germ cells (Fig 4F, right panels). Taken together, these results indicate that while the *Brca2*^*G25R/Ko*^*;Palb2*^*Ko/+*^ males are not lacking germ cells, there is an apparent failure of germ cell expansion. Overall, the significant enhancement of the mutant phenotype of *Brca2*^*G25R/Ko*^ males on a *Palb2*^*Ko/+*^ background strongly suggests that *Palb2* heterozygosity has a synergistic effect on the *Brca2*^*G25R*^ mutation.

### Decreased tumor latency in *Brca2*^*G25R*^ and *Palb2*^*Ko*^ mice

**Fig 5:**
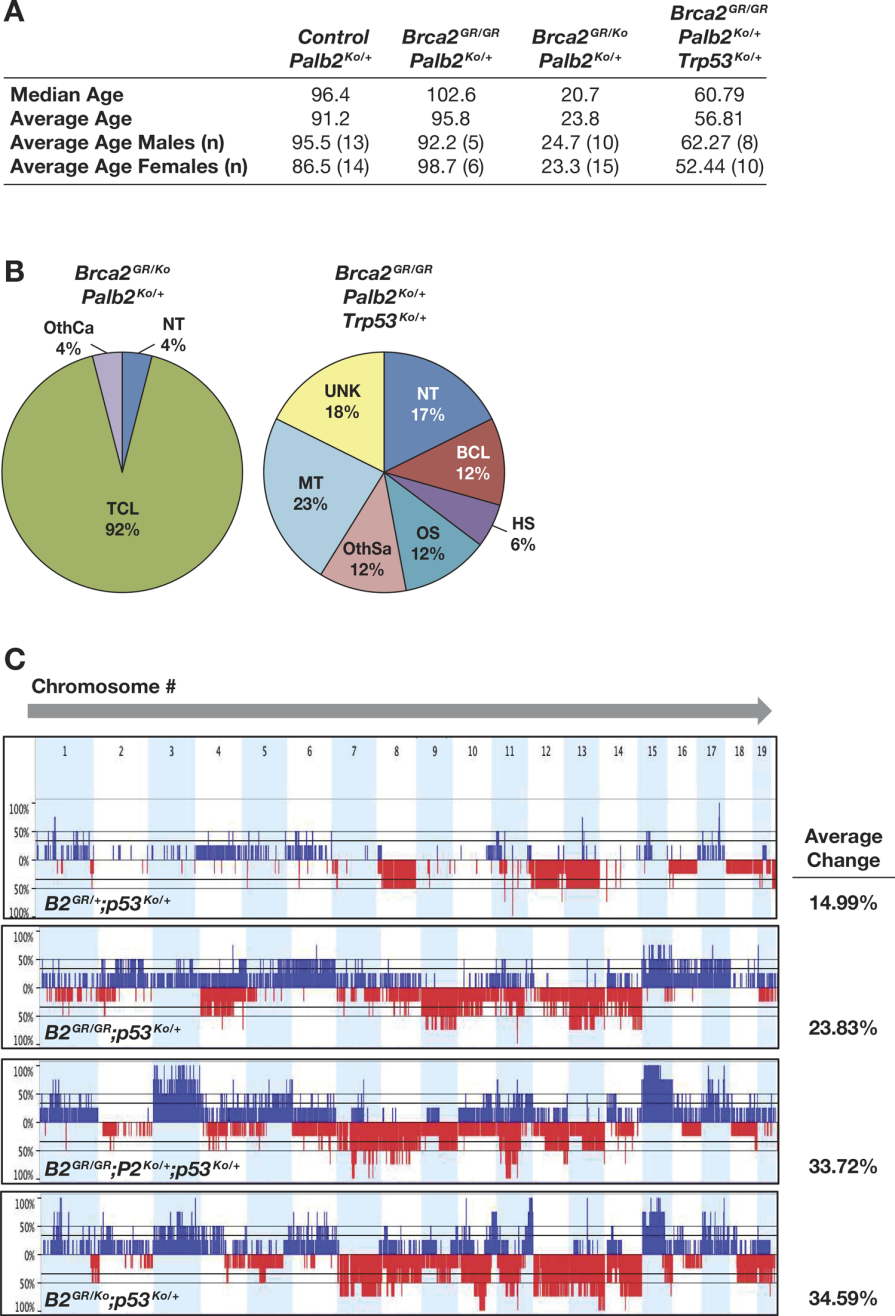
Increased tumor formation in *Brca2*^*G25R*^ mice in a *Palb2*^*Ko/+*^ genetic background. **A.** Median and average age of mice, including the number of animals for each genotype. **B.** Distribution of tumors found in the of *B2*^*GR/Ko*^*;P2*^*Ko/+*^ and *B2*^*GR/GR*^*;P2*^*Ko/+*^ mutant mice. Tumor abbreviations: NT = No tumor observed, BCL = B-cell lymphoma, TCL = T-cell lymphoma, HS = Histiocytic sarcoma, OS = Osteosarcoma, HemS = Hemangiosarcoma, OthSa = Other sarcomas, AD = Adenomas, OthCa = Other carcinomas, MT = Multiple tumors, UNK = cause of death unknown. **C.** Array CGH analysis of tumors from mice of indicated genotypes. These show the percentage of any known copy number variation within all tumors of the given genotype. Numbers at the top indicate the chromosome numbers. Blue lines represent gains and red lines represent losses. Controls are represented by either: *Brca2*^*+/+*^, *Brca2*^*Ko/+*^, or *Brca2*^*G25R/+*^ (with or without *Palb2*^*Ko/+*^ and/or *Trp53*^*Ko/+*^). Abbreviations: *Brca2*^*G25R/+*^ = ***B2***^***GR/+***^, *Brca2*^*G25R/G25R*^ = ***B2***^***GR/GR***^, *Brca2*^*G25R/Ko*^ = ***B2***^***GR/Ko***^, *Palb2*^*Ko/+*^ = ***P2***^***Ko/+***^, *Trp53*^*Ko/+*^ = ***p53***^***Ko/+***^.

We examined the effect of *Palb2* heterozygosity on the tumor predisposition of *Brca2*^*G25R/Ko*^ animals that had a mean survival time of 80 weeks (Fig 3A). Consistent with the significant effect on the fertility of these animals, the *Brca2*^*G25R/Ko*^;*Palb2*^*Ko/+*^ animals (n = 25, p<0.001) exhibited a mean survival time of 24 weeks with the majority of the animals (92%) succumbing to T-cell lymphoblastic lymphomas (Fig 3B, Fig 5A, Fig 5B, S6D Fig). Although the effect of *Palb2* heterozygosity on the tumor predisposition of *Brca2*^*G25R/G25R*^ animals is not any different from the control animals (mean survival: 96 weeks, n = 11, S6B and S6C Fig), on a *Trp53*^*Ko/+*^ genetic background the *Brca2*^*G25R/G25R*^;*Palb2*^*Ko/+*^ mice showed a significant decrease in survival compared to the control (57 week vs. 70 weeks, p = 0.018, Fig 3C). These mice developed diverse neoplasias; 12% had lymphomas (B-cell), 12% osteosarcomas, 18% other carcinomas, with 23% of the animals having multiple tumors (Fig 5A, Fig 5B, S6D Fig). The decrease in survival and increase in tumor predisposition of *Brca2*^*G25R*^ mutant mice on *Palb2*^*Ko/+*^ genetic background provides strong evidence to suggest that normal levels of interaction between BRCA2 and PALB2 is essential for tumor suppression.

### Degree of genomic instability in *Brca2*^*G25R/Ko*^*;Trp53*^*Ko/+*^ tumors is similar to *Brca*^*G25R/G25R*^*;Palb2*^*Ko/+*^*;Trp53*^*Ko/+*^ tumors

The difference in the tumor latency observed in mice of various genotypes, especially the significantly accelerated tumor formation in *Brca2*^*G25R/Ko*^*;Trp53*^*Ko/+*^ mice was correlated with the degree of genomic instability in the tumors. We examined the genomic profile of tumors by array comparative genomic hybridization (aCGH) analysis using an Agilent SurePrint G3 Mouse CGH Array (4X180K) chip. We examined tumors from *Brca2*^*G25R/+*^, *Brca2*^*G25R/G25R*^, *Brca2*^*G25R/Ko*^ mice, all on *Trp53*^*Ko/+*^ genetic background. Based on the total region affected by copy number variation (CNV), we observed a mean genomic change of 14.99% in *Brca2*^*G25R/+*^*;Trp53*^*Ko/+*^ tumors (CNV average, 121, Fig 5C, S7 Fig, S8 Fig). The *Brca2*^*G25R/G25R*^*;Trp53*^*Ko/+*^ tumors had an average genomic alterations of 23.83% (p = 0.23) with an average CNV of 443 (p = 0.054) (Fig 5C, S7 Fig, S8 Fig). The tumors from *Brca2*^*G25R/Ko*^*;Trp53*^*Ko/+*^ mice revealed the presence of even greater genomic alterations (34.6% p = 0.052) and also had a number of regions with gains and losses (CNV = 413, p = 0.068) (Fig 5C, S7 Fig, S8 Fig). These observations suggest that the mutation that affects the binding of BRCA2 with PALB2 is associated with increased genomic alterations in tumors. To further demonstrate that this genomic instability is due to disruption of the interaction between BRCA2 and PALB2, we examined the tumors from *Brca2*^*G25R/G25R*^*;Palb2*^*Ko/+*^*;Trp53*^*Ko/+*^ mice. These mice exhibited a marked reduction in the mean tumor latency compared to *Brca2*^*G25R/G25R*^*;Trp53*^*Ko/+*^ mice. When we examined tumors from these mice by aCGH analysis, we observed an increase in genomic instability from 23.8% to 33.7% (p = 0.07). These tumors had gains and losses similar to those seen in *Brca2*^*G25R/Ko*^*;Trp53*^*Ko/+*^ tumors and the total CNVs observed were 424(p = 0.036) (Fig 5C, S7 Fig, S8 Fig). Interestingly, one of the common gains observed in both the *Brca2*^*G25R/Ko*^*;Trp53*^*Ko/+*^ and *Brca2*^*G25R/G25R*^*;Palb2*^*Ko/+*^*;Trp53*^*Ko/+*^ tumors was on mouse chromosome 15 centered around the *Myc* locus (S8A Fig). Amplification of the region surrounding *Myc* is commonly seen in *BRCA2* tumors[30–33]. Taken together, these results demonstrate that the tumors that are genetically predicted to have the least interaction between BRCA2 and PALB2 have the highest levels of genomic instability.

### *Brca2*^*G25R*^ mouse embryonic fibroblasts (MEFs) under stress exhibit delayed cell cycle progression and decreased proliferation

To evaluate the effect of disruption of BRCA2-PALB2 interaction at the cellular level, we generated mouse embryonic fibroblasts from embryos of various genotypes. We evaluated the growth of these cells (using at least 2 independent lines from each genotype) from early passages. We found a significant reduction in the growth of mutant MEFs of various genotypes relative to the control MEFs (WT and *Palb2*^*Ko/+*^). The 2 lines that grew the slowest were *Brca2*^*G25R/Ko*^ and *Brca2*^*G25R/Ko*^*;Palb2*^*Ko/+*^ (S9A Fig, p<0.001 by day 5). To further evaluate the cause of this poor growth we examined the cell cycle profile of untreated and 100nM mitomycin C (MMC) treated MEFs. MMC is a DNA cross-linker that induces replication stress and leads to DSBs. There was no significant difference in the cell cycle profile of untreated MEF lines. Upon MMC treatment, we observed a modest increase in the number of cells at G2/M in *Brca2*^*Ko/+*^*;Palb2*^*Ko/+*^ (p = 0.028), *Brca2*^*G25R/Ko*^ (p = 0.022), *Brca2*^*G25R/G25R*^*;Palb2*^*Ko/+*^ (p = 0.016), and *Brca2*^*G25R/Ko*^*;Palb2*^*Ko/+*^ (p = 0.17) MEFs compared to the controls (S9C Fig). We evaluated the proliferative ability of these cells by BrdU incorporation and found no difference in any of the untreated MEFs. In response to 100nM MMC treatment, there was a significant decrease in BrdU incorporation in *Brca2*^*G25R/Ko*^ (p = 0.012) and *Brca2*^*G25R/Ko*^*;Palb2*^*Ko/+*^ MEFs (p = 0.009) (S9D and S9E Fig). Other lines were not statistically different from the controls. (S9D and S9E Fig) These results suggest that while there is persistent DNA damage in these cells upon MMC treatment, only the cells predicted to have the least interaction between BRCA2 and PALB2 exhibit a reduction in cell proliferation.

### *Brca2*^*G25R*^ MEFS exhibit genomic instability after MMC treatment

**Fig 6:**
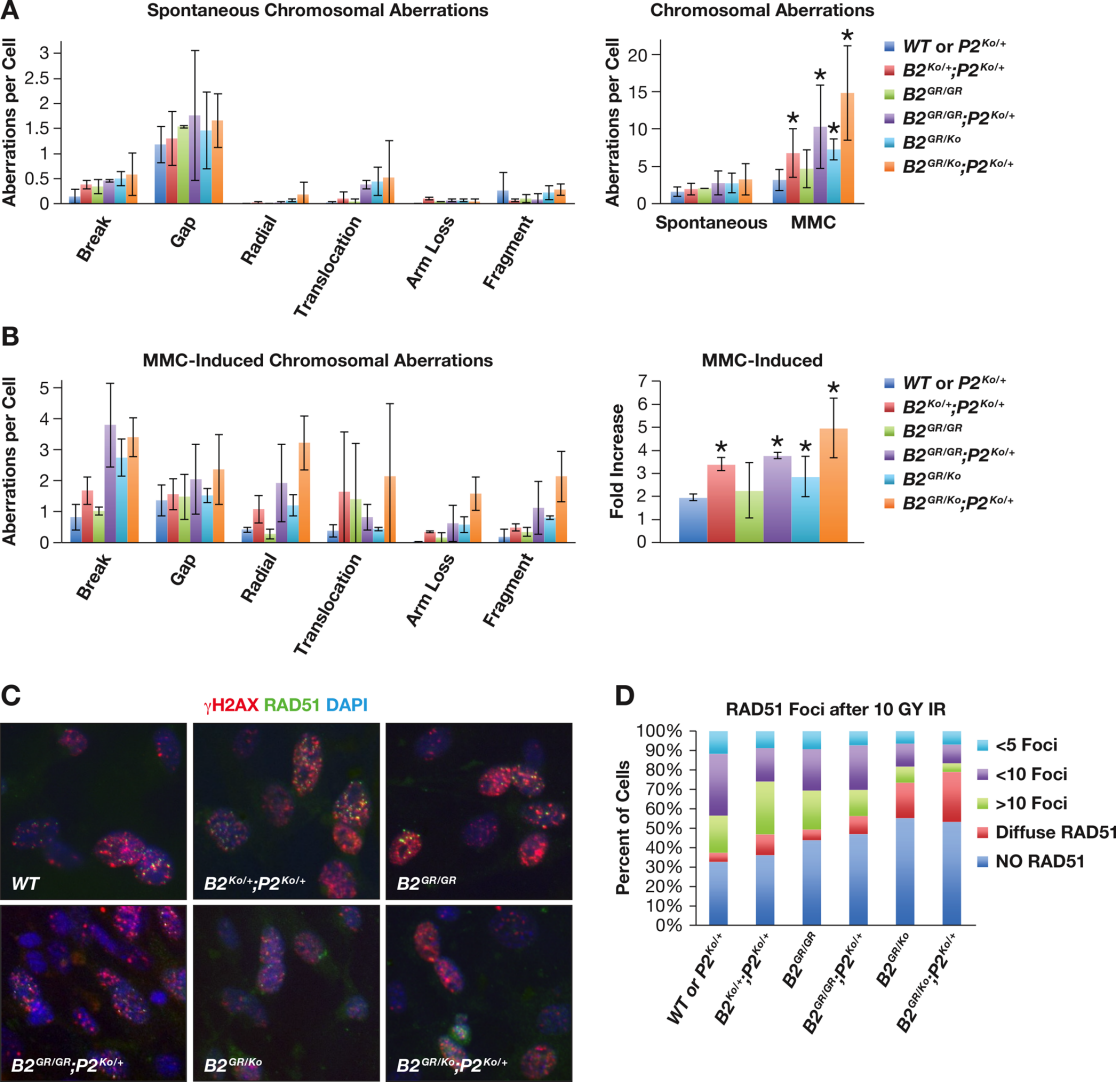
*Brca2*^*G25R*^ contributes to genomic instability and affects RAD51 foci formation. **A.** Spontaneous chromosomal aberrations seen in MEFs of various genotypes (left) and total chromosomal aberrations seen in untreated and 100nM MMC treated MEFs (right). **B.** Chromosomal aberrations seen with in MEFS of various genotypes treated with 100nM MMC (left) and fold increase in aberrations seen after 100nM MMC treatment (right). Error Bars: SD, * p<0.05 **C.** Representative images of RAD51 and γH2Ax foci formation after irradiation in primary MEFs of various genotypes. **D.** Quantification of RAD51 foci seen in cells that also have γH2AX foci. Controls are represented by: *Brca2*^*+/+*^ (with or without *Palb2*^*Ko/+*^). Abbreviations: *Brca2*^*+/+*^ = **WT,** *Brca2*^*Ko/+*^ = ***B2***^***Ko/+***^, *Brca2*^*G25R/G25R*^ = ***B2***^***GR/GR***^, *Brca2*^*G25R/Ko*^ = ***B2***^***GR/Ko***^, *Palb2*^*Ko/+*^ = ***P2***^***Ko/+***^.

With the *Brca2*^*G25R*^ mutant MEFs exhibiting delayed G2/M progression and reduced cell proliferation, we examined its effect on the genomic integrity of the cells. We examined metaphase spreads of untreated and MMC treated cells. We found very few chromosomal aberrations in untreated control MEFs (1.6–2.06 aberrations (ab)/cell). We observed a slight increase in breaks and translocations in *Brca2*^*G25R/G25R*^*;Palb2*^*Ko/+*^(2.76 ab/cell), *Brca2*^*G25R/Ko*^(2.74ab/cell), and *Brca2*^*G25R/Ko*^*;Palb2*^*Ko/+*^ (3.26ab/cell) (p = 0.0098, 0.0007, and 0.0001 respectively) (Fig 6A). After 100nM MMC treatment, *Brca2*^*G25R/G25R*^*;Palb2*^*Ko/+*^ (10.32 ab/cell), *Brca2*^*G25R/Ko*^,(7.28 ab/cell) and *Brca2*^*G25R/Ko*^*;Palb2*^*Ko/+*^ (14.84 ab/cell) MEFs showed a marked increase (3.8 fold, 2.9 fold, and 5.1 fold, respectively) in aberrations (breaks, radials, fragments and translocations) over controls MEFs (3.18–4.68 ab/cell) (Fig 6B, p<0.0001 for these three lines). Surprisingly, we also observed a 3.4 fold increase in breaks, radials, and translocations in *Brca2*^*Ko/+*^*;Palb2*^*Ko/+*^ MEFs (6.78 ab/cell) (Fig 6B, p = 0.0003). Taken together, these results show that BRCA2-PALB2 interaction is essential for maintenance of the genomic integrity.

### Decrease in RAD51 foci formation in *Brca2*^*G25R*^ MEFs

To examine the effect of disruption of BRCA2-PALB2 interaction on RAD51 recruitment in mitotic cells, we analyzed RAD51 foci formation in response to radiation induced DSBs. We irradiated cells with 1000 rads and immunostained after 3 hrs with γH2AX and RAD51. The percentage of cells that were γH2AX positive were very similar between the genotypes. Yet, there was a significant reduction in the number of cells lacking RAD51 foci in *Brca2*^*G25R/Ko*^ (55% of cells) and *Brca2*^*G25R/Ko*^*;Palb2*^*Ko/+*^ (53% of cells) (both p<0.00001, Fig 6C and 6D) when compared to the controls (33% of the cells). *Brca2*^*G25R/G25R*^ cells also had an increase in the number of cells lacking foci, (44% of cells, p = 0.0483) along with *Brca2*^*G25R/G25R*^*;Palb2*^*Ko/+*^ (47% of cells, Fig 6C and 6D, p = 0.0015). Interestingly, although *Brca2*^*Ko/+*^*;Palb2*^*Ko/+*^ cells exhibited a defect in cell growth and a moderate increase in chromosomal aberrations, they had normal RAD51 foci formation (Fig 6C and 6D). There were several *Brca2*^*G25R/Ko*^ and *Brca2*^*G25R/Ko*^*;Palb2*^*Ko/+*^ cells that also showed diffused RAD51 staining (18% and 26% of cells respectively, Fig 6C and 6D, p<0.0001). It is possible that this represents the RAD51 that is relocalized into the nucleus, but is unable to form foci due to weakened BRCA2-PALB2 interaction. We also observed a modest increase in this diffuse staining in *Brca2*^*G25R/G25R*^*;Palb2*^*Ko/+*^ and *Brca2*^*Ko/+*^*;Palb2*^*Ko/+*^ MEFs (9% and 11%, Fig 6C and 6D, p = 0.034 and p = 0.0204). Consistent with these observations, we found a reduction in RAD51 foci in PCNA positive S-phase cells (S10A Fig). These results suggest that the mutation in the PALB2 binding domain decreases the ability of RAD51 recruitment.

### BRCA2-PALB2 interaction is essential for replication fork protection

**Fig 7:**
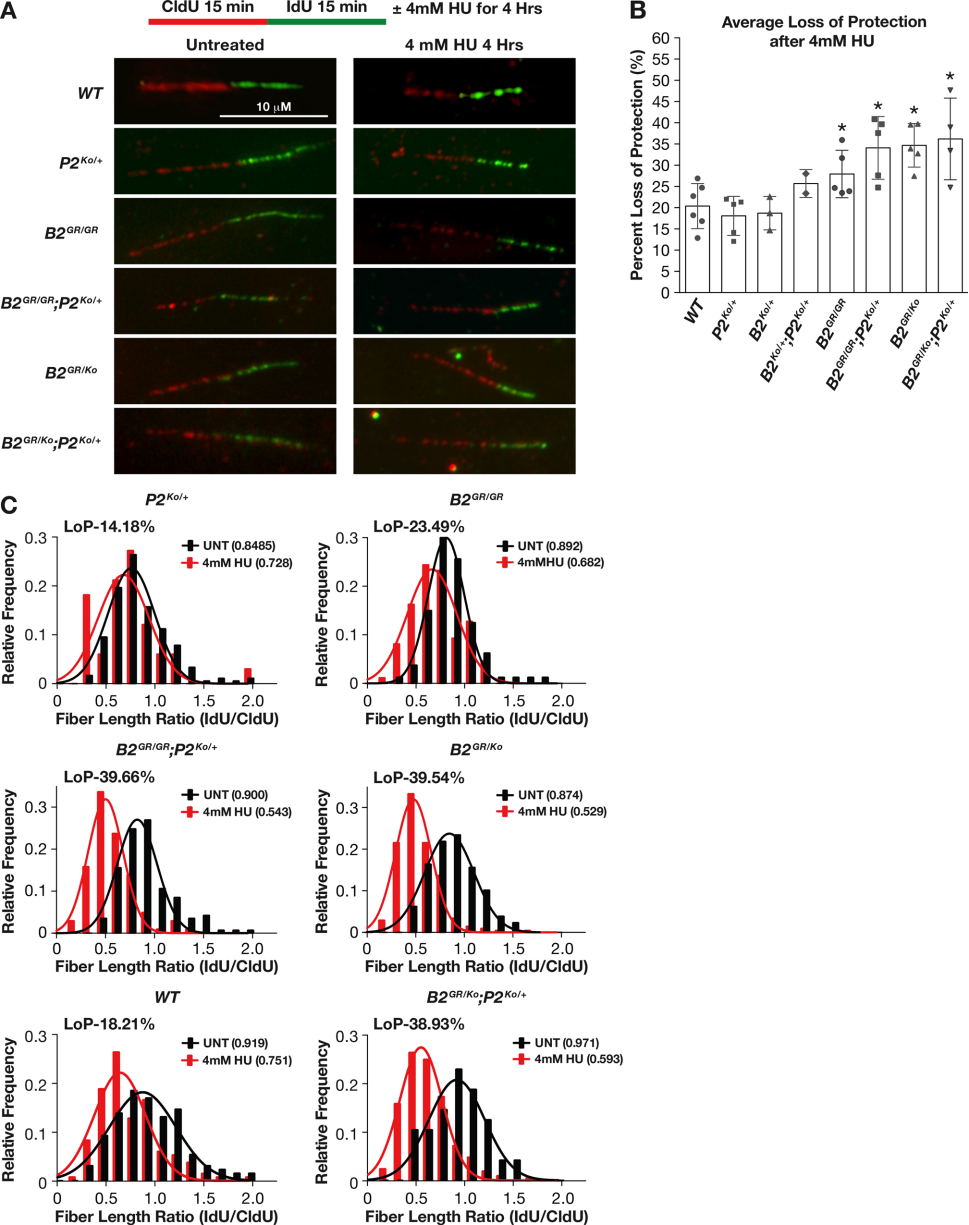
*Brca2*^*G25R*^ mutation affects protection of stalled replication forks. **A.** Representative images of the fork protection assay in primary MEFs of indicated genotypes in both untreated and 4mM HU treated conditions. The x-axis represents the fiber length ratio (IdU/CldU) and y-axis represents the frequency of the fibers that have the corresponding IdU/CldU ratio. **B.** Average loss of protection (LoP) for the various genotypes across multiple experiments. **C.** Representative frequency of IdU/CldU ratios in untreated (black) and HU treated (red) MEFs indicating the average ratio for untreated and HU treated (number in parenthesis) as well as percent of LoP. LoP = (1-*a*/*b*)X100, where *a* is the IdU/CldU ratio after HU treatment and *b* is the IdU/CldU ratio without HU treatment. Error bars: SD, * p<0.05. Controls are represented by: *Brca2*^*+/+*^, *Brca2*^*Ko/+*^, and *Palb2*^*Ko/+*^. Abbreviations: *Brca2*^*+/+*^ = **WT,** *Brca2*^*Ko/+*^ = ***B2***^***Ko/+***^, *Brca2*^*G25R/G25R*^ = ***B2***^***GR/GR***^, *Brca2*^*G25R/Ko*^ = ***B2***^***GR/Ko***^, *Palb2*^*Ko/+*^ = ***P2***^***Ko/+***^.

BRCA2 and PALB2 are essential for the protection of the nascent DNA strands at stalled replication forks [5, 34]. To examine if a physical interaction between these two proteins is essential for fork protection as in the case of DSB repair, we examined the integrity of the nascent DNA strands under replicative stress in the primary MEFs. We labeled the replicating strand of DNA first with thymidine analog, CldU (red), and then with thymidine analog, IdU (green), for 15 minutes each followed by hydroxyurea (HU) treatment (which induces replication stress by depleting the dNTP pool) for 4 hours (Fig 7A). We measured the length of green and red tracks and estimated the loss of fork protection based on the ratios of IdU/CldU in the presence and absence of HU treatment. Loss of protection represents the change in average fiber length in response to replicative stress compared to when there is no fork degradation. It was calculated as the percent reduction in the ratio of DNA fiber length after HU treatment and before HU treatment compared to when the fork is fully protected and the ratio is expected to be 1. In the control cells, wildtype (n = 6), *Brca2*^*Ko/+*^(n = 3), and *Palb2*^*Ko/+*^ (n = 5), we observed a mean decrease in protection of 19% (3 independent experiments) (Fig 7). The loss of fork protection in *Brca2*^*Ko/+*^*;Palb2*^*Ko/+*^ MEFs was increased to 26% (n = 2). The *Brca2*^*G25R/G25R*^ cells showed a 28% loss in fork protections (n = 5, p = 0.003), which increased significantly on a *Palb2*^*Ko/+*^ background (34%, n = 4, p<0.0001). Interestingly, the *Brca2*^*G25R/Ko*^ MEFs exhibited 35% loss of protection (n = 5, p<0.0001) however, did not show any further loss when one allele of *Palb2* was deleted (36%, n = 4) (Fig 7). While there is an apparent loss of fork protection with the disruption of BRCA2-PALB2 interaction, there seems to be a maximum loss that can be tolerated by the cells or it reflects the sensitivity of the DNA fiber assay. We also evaluated the ability of the DNA replication forks to restart after replication stress. We examined this in the MEFs that had exhibited the most severe cellular phenotype (*Brca2*^*G25R/KO*^*;Palb2*^*Ko/+*^). Control and mutant cells were first labeled with CldU, replication was then stalled with 0.5mM HU for 1.5hrs and subsequently resumed in the presence of IdU. These cells were then analyzed for the length of the CldU and IdU labeled DNA fibers to determine whether the stalled forks had restarted or there was a new origin of replication. Overall, here was no significant difference between control and *Brca2*^*G25R/KO*^*;Palb2*^*Ko/+*^ cells suggesting that fork restart was not affected in the mutant cells (S10B Fig). We conclude from these observations that similar to the role in HR-mediated DSB repair, BRCA2-PALB2 interaction is a major contributor to replication stress induced fork protection.

## Discussion

BRCA2 performs its function as a care-taker of the genome because of its ability to bind to a number of key proteins. One such binding partner is PALB2. Functional studies using knockout or knockdown of *PALB2* have demonstrated that it is essential for BRCA2-mediated recruitment of RAD51 as well as for protection of stalled replication forks (review [35]). While these studies show the importance of PALB2, they do not shed light on the relevance of a direct interaction between these two proteins. Although three mutations in the PALB2-binding domain (G25R, W31C and W31R) that disrupt the interaction between the two proteins are linked to breast cancer development, their precise disease association remains uncertain due to limited family-linkage data [21].

To address the significance of the interaction between the two proteins and examine the effect of disruption of this interaction on tumor development, we generated a novel *Brca2* knock-in mouse model carrying the G25R mutation, which results in DNA repair defects without affecting cell viability. Therefore, it provides a unique opportunity to dissect the relevance of the interaction between PALB2 and BRCA2 in various biological processes including tumorigenesis. We have generated mice of various genotypes, carrying one or two alleles of *Brca2*^*G25R*^. Furthermore, we have combined these genotypes with *Palb2* heterozygosity to reduce PALB2 levels to gradually and systematically reduce the interaction without affecting viability. These mice exhibit mutant phenotypes that become increasingly severe as the interaction between BRCA2 and PALB2 is gradually reduced.

### RAD51 recruitment and replication fork protection are impaired due to disruption of BRCA2-PALB2 interaction

Overall, *Brca2*^*G25R*^ mutation did not affect viability or fertility of *Brca2*^*G25R/ G25R*^ mice. However, *Brca2*^*G25R/Ko*^ males exhibited a delay in meiotic progression due to a defect in RAD51 recruitment in spermatocytes. This resulted in persistence of unrepaired DNA damage and eventual cell death causing loss of germ cells. Interestingly, on a *Palb2*^*Ko/+*^ background, while the *Brca2*^*G25R/G25R*^ mice did not exhibit any phenotypic defect, the *Brca2*^*G25R/Ko*^ mice were relatively smaller in size and the males were infertile. The testes of *Brca2*^*G25R/Ko*^*;Palb2*^*Ko/+*^ males revealed the presence of germ cells at 3 weeks of age. However, germ cells were lost by 7 weeks of age leading to a germ cell-less phenotype. A lack of male germ cells has been previously reported in mice expressing a hypomorphic allele of *Brca2* encoding a truncated protein but the timing or the mechanism of germ cell loss has not been described [36, 37].

Similar to the spermatocytes, a defect in PALB2-BRCA2 interaction also affects RAD51 foci formation in response to IR-induced DSBs in somatic cells. *Brca2*^*G25R/Ko*^ and *Brca2*^*G25R/Ko*^*;Palb2*^*Ko/+*^ MEFs exhibited a significant reduction in RAD51 foci suggesting that the interaction between BRCA2 and PALB2 is essential for proper recruitment of RAD51 to the site of DSB.

Recent studies have suggested that heterozygosity of *BRCA2* as well as *PALB2* results in some loss of DNA replication fork protection and a defect in DNA damage response, which contributes to genomic instability [38, 39]. However, in the present study, single heterozygous mice and MEFs (*Brca2*^*Ko/+*^, *Brca2*^*G25R/+*^, and *Palb2*^*Ko/+*^) did not reveal any overt defects. We observed a small loss of fork protection in *Brca2*^*G25R/G25R*^ and *Brca2*^*Ko/+*^*;Palb2*^*Ko/+*^ MEFs but *Brca2*^*G25R/G25R*^*;Palb2*^*Ko/+*^, *Brca2*^*G25R/Ko*^ and *Brca2*^*G25R/Ko*^*;Palb2*^*Ko/+*^ MEFs exhibited a significant fork degradation. Our results show that although both BRCA2 and PALB2 have their own DNA binding motifs, as well as independent RAD51 binding motifs, their interaction is essential for proper recruitment of RAD51 to the sites of DNA damage and protection of stalled replication forks.

### Genomic instability and tumor susceptibility due to disruption of BRCA2-PALB2 interaction

As observed in the MEFs derived from mice of various genotypes with mutations in *Brca2* and *Palb2*, a defect in RAD51 recruitment as well as a loss of protection of the replication fork leads to genomic instability. Based on the tumor predisposition of these mice, it is evident that the genomic instability contributes to tumorigenesis. *Brca2*^*G25R/Ko*^ mice had a significantly reduced tumor free survival compared to *Brca2*^*G25R/+*^ as well as *Brca2*^*G25R/G25R*^ mice, which is further enhanced on a *Trp53*^*Ko/+*^ genetic background. Their tumor predisposition is reminiscent of the *Brca2* mutant mice that lack exon 27 (*Brca2*^*Δ27/Δ27*^), in terms of latency, frequency of tumor formation and enhancement on a *Trp53*^*Ko/+*^ genetic background [28, 40, 41]. However, the *Brca2*^*G25R/Ko*^ animals predominately developed osteosarcomas on the *Trp53*^*Ko/+*^ background, unlike the *Brca2*^*Δ27/Δ27*^*;Trp53*^*Ko/+*^ mice that developed a variety of tumor types. A recent sequence analysis of the exome of osteosarcomas reported increased incidence of *BRCA2* mutations as well as signatures associated with BRCA2 loss suggesting the involvement of BRCA2 in the development of osteosarcomas [42–44]. The *Brca2*^*G25R/Ko*^;*Palb2*^*Ko/+*^ animals that result in the least interaction between BRCA2 and PALB2 and exhibited the shortest tumor latency, predominantly developed lymphoblastic T-cell lymphomas, similar to the mice carrying a truncating mutation in exon 11 of *Brca2* [36, 37].

Overall, the tumor incidence and tumor latency in mice for the most part correspond with the degree of genomic instability observed in the MEFs. However, there were some exceptions. For example, the *Brca2*^*G25R/G25R*^ MEFs exhibited a mild defect in RAD51 recruitment and fork protection but the survival of mice was not affected except they had a mild increase in tumor formation at 24 months of age. The tumor free survival of these mice was also not altered on a *Trp53*^*Ko/+*^ genetic background.

**Fig 8:**
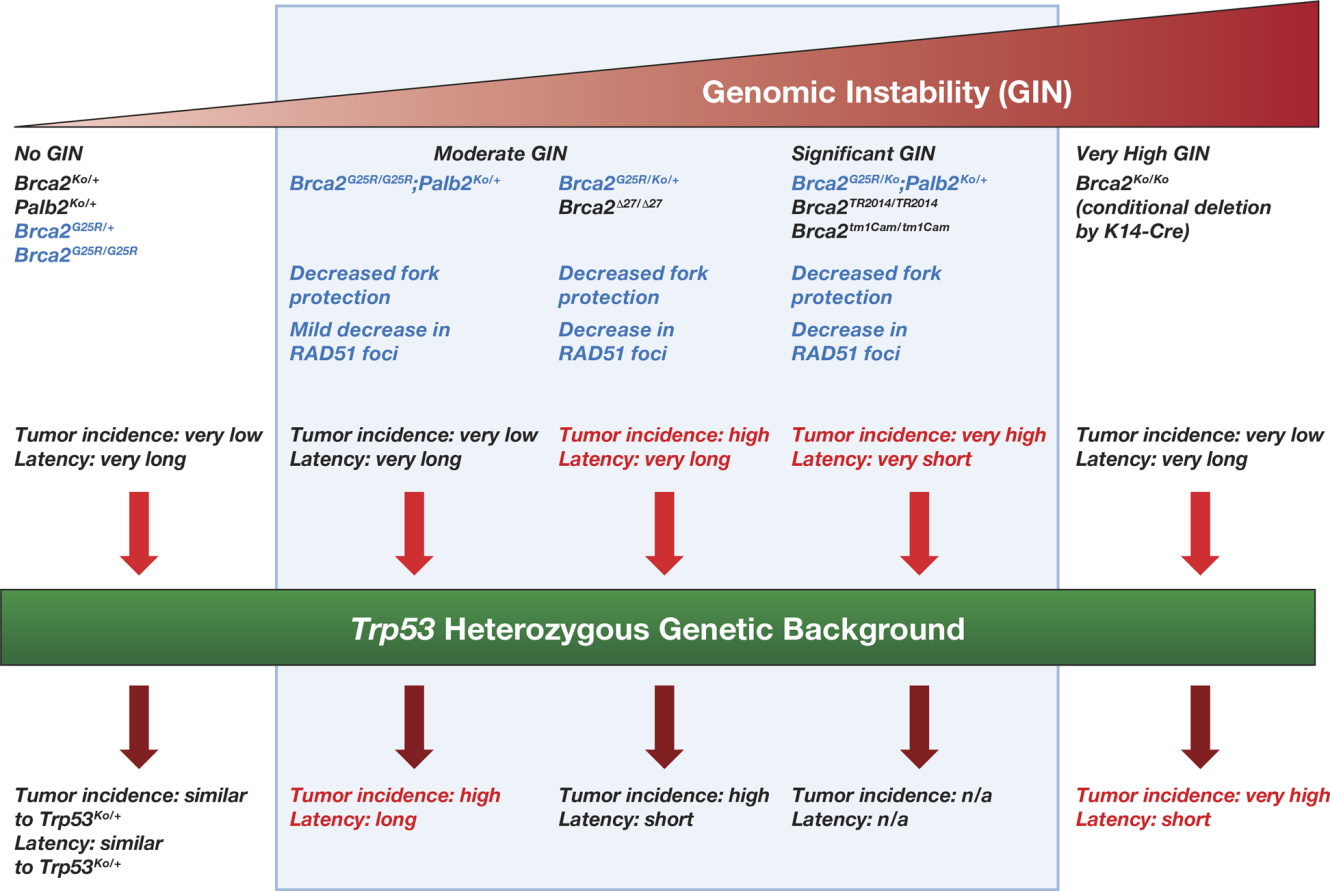
Relationship between genomic instability and tumorigenesis. Mutations in *Brca2* that result in mild genomic instability (GIN) do not contribute to tumorigenesis. Mutations that result in moderate GIN lead to tumor formation in mutant mice. However, mutations (e.g. functionally null allele, *Brca2*^*Ko*^*)* that cause severe GIN result in increased cell death and decreased tumor formation. Genotypes in blue indicate the mice described in the present study.

While an increase in genomic instability correlates well with an increase in tumor susceptibility, having a significantly high genomic instability may not necessarily enhance tumorigenesis (Fig 8). When *Brca2* is rendered functionally null by CRE-mediated recombination in the epithelial cells using cytokeratin K14 promoter (which is expressed during embryogenesis), tumor incidence is low and tumor latency is very long [45]. Although the effect of germline inheritance of the mutant alleles in all cell lineages (as in the present study) versus loss in specific cell lineage cannot be ruled, the longer latency is believed to be due to the strong apoptotic response induced by the high genomic instability in cells lacking BRCA2 [46]. This is supported by the observation that the tumor incidence is increased and the tumor latency is significantly reduced when apoptosis is suppressed on a *Trp53* heterozygous background [45]. The most striking phenotype in this present study is the high tumor incidence in *Brca2*^*G25R/Ko*^ as well as *Brca2*^*G25R/Ko*^*;Palb2*^*Ko/+*^ mice with wild-type *Trp53*. Sixty-nine percent of *Brca2*^*G25R/Ko*^ and 100% of *Brca2*^*G25R/Ko*^*;Palb2*^*Ko/+*^ mice developed tumors, with an average latency of 82 and 24 weeks, respectively. Considering these genotypes have no effect on the viability of mice, the genomic instability induced in cells must not be high enough to induce a strong apoptotic response but it must be significant enough to result in cellular transformation and tumorigenesis. Similar high tumor incidence has been reported in other mice carrying *Brca2* hypomorphic alleles that result in viable mice, such as *Brca2*^*tm1Cam*^, *Brca2*^*Tr2014*^ [36, 37]. Our findings suggest that while complete loss of BRCA2 is clearly associated with increased risk of breast, ovarian and other cancers, hypomorphic alleles that disrupt key BRCA2 functions, are likely to be equally pathogenic. It is possible that such mutations may cause cancer in carriers at a younger age because they are not dependent on an additional mutation in *TRP53* or other genes that have an anti-apoptotic response.

In conclusion, our results provide strong evidence to support the importance of a physical interaction between BRCA2 and PALB2. In spite of their ability to independently bind to RAD51, their heterodimerization via the N-terminus of BRCA2 and the C-terminus of PALB2 is essential for their function. Disruption of this interaction not only affects the DSB repair but also the ability to protect the stalled replication forks. These unique mouse models will help distinguish the importance of HR and fork protection in BRCA2’s and PALB2’s ability to protect genomic integrity. In addition, the unique mouse models described here will be useful for preclinical studies because of their high tumor incidence and low tumor latency.

## Materials and Methods

### Ethics statement

Mice were treated in accordance with the recommendations in the Guide for the Care and Use of Laboratory Animals (The National Academies Press; Washington DC, 8th edition). The protocol was approved by the Animal Care and Usage Committee of NCI-Frederick (NIH) (Animal Study Proposals: 12–471 and 15–471).

### Generation of G25R knock-in targeting vector

A single base substitution (G>A) was generated in the 6^th^ nucleotide of exon 3 to change codon 25 (GGA> AGA, Gly25Arg) of *Brca2* clone in BAC RPCI22-421-23A by recombineering in SW102 cells using the *galK-*based selection-counterselection method as described previously[21, 47]. The G>A substitution generated a *Mse*I restriction enzyme recognition site. Next, a *loxP*-PGK-Tn5-*neomycin*-bp(A)-*loxP* cassette along with *Ban*II and *Eco*RI restriction sites were inserted into intron 3 by recombineering. Finally, a 10.7 kb genomic region of *Brca2* was retrieved by gap repair from the BAC into pBSK(+) plasmid containing the *Thymidine Kinase* (TK) gene under the control of the MC1 promoter. The resultant plasmid was the G25R knock-in targeting vector (S1 Fig).

### Targeting *Brca2* in ES cells and generation of *Brca2*^*G25R-Neo/+*^ mice

G25R knock-in targeting vector was linearized with *Not*I and electroporated into V6.4 (129 X C57BL/6J) ES cell line as described previously [48]. G418^R^, FIAU^R^ ES cell clones were screened by Southern analysis of *Ban*II and *Eco*RI restriction enzyme digested genomic DNA using 5’ and 3’ probes (S1 Fig). A few correctly targeted ES cell clones were injected into C57BL/6 blastocysts to generate chimeras. One of these chimeras transmitted the targeted allele in the germ line and *Brca2*^*G25R-Neo/+*^ pups were obtained.

### Mice

To obtain *Brca2*^*G25R/+*^ mice, *Brca2*^*G25R-Neo/+*^ mice were crossed to β-actin-Cre-deleter strain [49]. To generate *Palb2*^*Ko/+*^ mice, CG0691 ES cells obtained from SIGTR were injected to into C57BL/6 blastocysts. Chimeras were crossed with C57BL/6 females to *Palb2*^*Ko/+*^ pups. *Brca2*^*G25R/Ko*^ were obtained by breeding *Brca2*^*G25R/G25R*^ or *Brca2*^*G25R/+*^ mice with *Brca2*^*ko/+*^ mice [50]. *Brca2*^*G25R/Ko*^ mice were crossed with *Palb2*^*Ko/+*^ and *Trp53*^*Ko/+*^ mice to obtain mice of various genotypes used in the study. Mouse colony was maintained on a mixed C57BL/6 and 129/SvEv genetic background. All animal studies were performed as per the protocols outlined in the Guide for the Care and Use of Laboratory Animals and approved by the NCI-Frederick Animal Care and Use Committee.

### Genotyping

For genotyping purposes, genomic DNA was extracted from tail biopsies, fresh or frozen tumor tissues following standard procedures. Genotyping was performed by PCR. Briefly, approximately 50 ng of DNA was amplified with the Taq DNA polymerase using primers listed in Supplementary Information (S1A and S1D Fig, S1 Table).

### Histology and immunohistochemistry

For histology, tissues were fixed in 10% formalin overnight, paraffin-embedded, sectioned and stained with H&E (hematoxylin and eosin). Statistical differences in tumor types were assessed via Fisher's exact test, and survival curves were analyzed utilizing Prism Graphpad. For germ-cell visualization 3-week old testis sections were immunostained with germ cell marker (MVH) as previously described [51]. Antibodies: Rabbit anti-DDX4/MVH (Abcam ab13840; 1:400); goat anti-rabbit Alexa 488 conjugate (Molecular Probes A11008; 1:1,000).

### Mouse embryonic fibroblasts

MEFs were generated from E13.5 embryos and cultured in 10% FBS at 5% O_2_. Cell growth analyses were done by seeding 10cm plates with 5 X 10^5^ cells and counting the cells every day. Metaphases were collected after a 24 hour treatment with 100nM MMC and spread as previously described [52]. Immunofluorescence of RAD51 foci was done by seeding cells on coverslips, irradiating with 1000 rads the following day. Fixed cells were collected after 3 hours and cells were stained with Mouse anti-γH2AX (1:500, Millipore JBW301) and rabbit anti-RAD51 (1:250, Millipore PC130).

### TUNEL staining

Paraffin sections of testes were TUNEL stained using the In Situ Cell Death Detection Kit (Roche 11684817910) following the directions included with the kit.

### Meiotic chromosome analysis

Meiotic spreads were performed as previously described[53]. Primary antibodies used in this study: rabbit anti-SYCP3 (1:500, Abcam ab13840); mouse anti-γH2AX (1:500, Millipore JBW301); mouse anti-SYCP3 (1:500 Abcam ab20244); rabbit anti-RAD51 (1:250, Millipore PC130).

### CGH array

A comparative genomic hybridization array was used for 4 tumors from the following genotypes, *Brca2*^*G25R/+*^*;Trp53*^*Ko/+*^ (2 Lymphomas, 2 Osteosarcoma), *Brca2*^*G25R/G25R*^*;Trp53*^*Ko/+*^, (2 Lymphomas, 2 Osteosarcoma) *Brca2*^*G25R/Ko*^*;Trp53*^*Ko/+*^ (4 Osteosarcomas) and *Brca2*^*G25R/G25R*^*;Palb2*^*Ko/+*^*;Trp53*^*Ko/+*^ (2 Sarcomas, 2 Osteosarcoma). The DNA was isolated using the Qiagen DNeasy Blood and Tissue Kit, wild-type mouse tail DNA was used as the control. The DNA was hybridized to an Agilent SurePrint G3 Mouse CGH Array 4X180K chip. The results from the array were analyzed using Nexus Copy Number from BioDiscovery.

### DNA fibers

DNA fiber analysis was performed as previously described with minor modifications [54, 55]. Briefly, cells were seeded at 50% on the previous day. CldU was added to media for 15 min, cells were washed 3X with pre-warmed 1X PBS. Then IdU was added in pre-warmed media for 15 min. Cells were then treated with 4mM HU for 4 hrs. Cells were washed 2X with pre-warmed 1X PBS, trypsinized and placed in cold PBS at a concentration of 2.5 X 10^5^ cells/ml. On pre-cleaned slides 7.5 μL of cell lysis solution (200mM Tris-HCl pH 7.4, 50mM EDTA, 0.5%SDS) was placed near the top of the slide. Then 2.5 μL of cells were added to this drop, mixed and then left at RT for 8 minutes. The slide was then tilted to allow spreading of fibers at approximately 45° angle to allow the drop to slowly run down the slide. The slides were then air-dried. Next, the slides were fixed in methanol/acetic acid 3:1 at 4° O/N. Slides were then re-hydrated with 1X PBS, denatured in 2.5M HCL for 1 hr, washed 5X with PBS. The slides were then blocked (2% BSA, 0.1% Tween 20, 1X PBS) for 40 minutes. The slides were then labeled with mouse anti-BrdU/IdU 1:100 (Becton Dickinson, #347580) and rat anti-BrdU/CldU (1:500) for 2.5hrs at RT or 4° O/N. Slides were then washed 5X with 0.2% PBS-Tween 20. Secondary antibodies, Alexa-fluors anti-rat 594, and anti-mouse 488 were used at 1:300 for 1 hr in dark at RT. Slides were washed again with 0.2% PBST, air-dried and mounted with anti-fade. Fibers were then visualized and imaged at 63X. Measurements of fibers were performed utilizing ImageJ software.

## Supporting Information

S1 Fig Gene targeting Scheme and genotyping of *Brca2*^*G25R*^. **A:** Schematic representation of the gene targeting strategy to generate the *Brca2*^*G25R*^ knock-in allele showing the wild type locus (*Brca2*^*WT*^), targeting vector, targeted allele (*Brca2*^*G25R-Neo*)^ showing the presence of *loxP-Neo-loxP* cassette and the *Brca2*^*G25R*^ knock-in allele with the point mutation and a single *loxP* site. The first four exons of *Brca2* exons are indicated as boxes with corresponding numbers. Location of restriction sites used for Southern-based genotyping are indicated by B (*Ban*II) and E (*Eco*RI). G25RGenoF and G25RGenoR designate location and direction of primers (in blue) used for PCR-based genotyping. Asterisk marks the location of the G>A mutation in the first base of codon 25 of *Brca2* in exon 3. Location of probes used to detect 5′ and 3′ end targeting are shown with a solid box below the *Brca2*^*WT*^ locus. **B.** Southern blot analysis showing correct gene targeting. **D.** Sequence read confirming G>A substitution in codon 25 of *Brca2*. **D.** Schematic representation of the *Brca2* null allele (*Brca2*^*Ko/+*^) showing deletion of a portion of exon 11 which is replaced with human *HPRT1* minigene. **E.** PCR-based genotyping of *Brca2*^*G25R*^ allele utilizing the primers show in blue arrows in **A.**
(PDF)

S2 Fig RAD51 Foci is decreased on the sex body at Pachynema. **A.** Representative meiocytes at pachynema of (*B2*^*Ko/+*^, left and *B2*^*GR/Ko*^, right) stained with SYCP3 (top, red), and RAD51 (middle, green) and merged (Bottom), arrows indicate the XY Body. **B.** Quantification of RAD51 foci along the XY body. Error bars: SD, * p<0.05. Controls are represented by either: *Brca2*^*Ko/+*^ or *Brca2*^*G25R/+*^. Abbreviations: *Brca2*^*Ko/+*^ = ***B2***^***Ko/+***^, *Brca2*^*G25R/+*^ = ***B2***^***GR/+***^, *Brca2*^*G25R/G25R*^ = ***B2***^***GR/GR***^, *Brca2*^*G25R/Ko*^ = ***B2***^***GR/Ko***^.
(PDF)

S3 Fig Increased Apoptosis at 7 weeks leads to decreased testis size of *Brca2*^*GR/Ko*^ males. Representative images of testes with TUNEL staining (green) and counterstained with DAPI (blue) to show the cells that are undergoing apoptosis at 3 weeks (**A.**) and 7 weeks (**B.**). Quantification of TUNEL positive cells at 3 weeks (**C.**) and 7 weeks (**D.**). Error Bars: SD, *p<0.05.
(PDF)

S4 Fig Tumor predisposition of *Brca2*^*G25R*^ mice by gender. Distribution of tumor types found in mice by gender of indicated genotypes. **A.** Females and **B.** Males. Abbreviations: NT = No tumor observed, BCL = B-cell lymphoma, TCL = T-cell lymphoma, HS = Histiocytic sarcoma, OS = Osteosarcoma, HemS = Hemangiosarcoma, OthSa = Other sarcomas, AD = Adenomas, OthCa = Other carcinomas, MT = Multiple tumors.
(PDF)

S5 Fig Tumor predisposition of *Brca2*^*G25R*^ mice by gender in *Trp53*^*Ko/+*^ background. Distribution of tumor types found in mice of indicated genotypes. **A.** Females and **B.** Males. Abbreviations: NT = No tumor observed, BCL = B-cell lymphoma, TCL = T-cell lymphoma, HS = Histiocytic sarcoma, OS = Osteosarcoma, HemS = Hemangiosarcoma, OthSa = Other sarcomas, AD = Adenomas, OthCa = Other carcinomas, MT = Multiple tumors, UNK = cause of death unknown.
(PDF)

S6 Fig *Brca2*^*GR*^*; Palb2*^*Ko/+*^ tumor predisposition and tumor distribution. **A.** Testis cross section of *Brca2*^*Ko/+*^*;Palb2*^*Ko/+*^ mice showing normal spermatogenesis at 3 weeks and 7 weeks of age. Far left panel: Testis cross section of *Brca2*^*GR/GR*^*;Palb2*^*Ko/+*^ mouse at 25 weeks showing normal spermatogenesis. **B.** Kaplan-Meier tumor-free survival curves of *Control*, *Control;Palb2*^*Ko/+*^ and *Brca2*^*GR/GR*^*;Palb2*^*Ko/+*^ mutant animals (104 week study). **C.** Total distribution of tumors found in indicated genotypes. **D.** Tumor distribution broken down by gender of indicated genotypes. Abbreviations: NT = No tumor observed, BCL = B-cell lymphoma, TCL = T-cell lymphoma, HS = Histiocytic sarcoma, OS = Osteosarcoma, HemS = Hemangiosarcoma, OthSa = Other sarcomas, AD = Adenomas, OthCa = Other carcinomas, MT = Multiple tumors, UNK = cause of death unknown. Controls are represented by: *Brca2*^*+/+*^ and *Brca2*^*Ko/+*^. Abbreviations: *Brca2*^*G25R/G25R*^ = ***B2***^***GR/+***^, *Palb2*^*Ko/+*^ = ***P2***^***Ko/+***^.
(PDF)

S7 Fig aCGH analysis reveals increased genomic instability in *Brca2*^*G25R*^ individual tumors. Individual copy number variation (CNV) graphs for each of the heterogeneous tumors across the genome. Chromosome number is at the bottom and amount of gain of loss is indicated at the Y-axis. (+1 or -1 indicates a 2 copy gain or 2 copy loss) Abbreviations: Ly = Lymphoma, OS = Osteosarcoma, HS = Histiocytic Sarcoma, Sarc = Sarcoma.
(PDF)

S8 Fig aCGH analysis reveals increased genomic instability in *Brca2*^*G25R*^ tumors. **A.** Image of amplified region around the *myc* locus in *Brca2*^*G25R/G25R*^ *Palb2*^*Ko/+*^ and *Brca2*^*G25R/Ko*^ tumors on a *Trp53*^*Ko/+*^ genetic background. **B.** Percent genome change, and **C.** total number of copy number changes in the individual tumors. Controls are represented by: *Brca2*^*G25R/+*^; *Trp53*^*Ko/+*^. Abbreviations: *Brca2*^*G25R/+*^ = ***B2***^***GR/+***^, *Brca2*^*G25R/G25R*^ = ***B2***^***GR/GR***^, *Brca2*^*G25R/Ko*^ = ***B2***^***GR/Ko***^, *Palb2*^*Ko/+*^ = ***P2***^***Ko/+***^, *Trp53*^*Ko/+*^ = ***p53***^***Ko/+***^.
(PDF)

S9 Fig Mouse embryonic fibroblast analysis reveals decreased cell growth, impaired cell cycle, and decreased proliferation. **A.** Fold change of cell growth over the course of 5 days of MEFs of various genotypes. **B.** Untreated and 100nM MMC treated cell cycle profiles of MEFs of the various genotypes. **C.** BrdU incorporation of untreated and 100nM MMC treated MEFs of the various genotypes. Error Bars: SD, *p<0.05. Controls are represented by: *Brca2*^*+/+*^ (with or without *Palb2*^*Ko/+*^). Abbreviations: *Brca2*^*+/+*^ = **WT,** *Brca2*^*Ko/+*^ = ***B2***^***Ko/+***^, *Brca2*^*G25R/G25R*^ = ***B2***^***GR/GR***^, *Brca2*^*G25R/Ko*^ = ***B2***^***GR/Ko***^, *Palb2*^*Ko/+*^ = ***P2***^***Ko/+***^.
(PDF)

S10 Fig *Brca2*^*G25R*^ MEFs have decreased RAD51 foci in S-Phase, yet still maintain the ability to restart stalled replication forks. **A.** RAD51 foci quantification in cells that are PCNA positive in indicated genotypes. **B.** Fork restart assay: The cells were pulsed with CldU for 15 minutes, then 0.5mM HU was added for 1.5 hrs to stall replication and then released into media containing IdU for 15 minutes. CldU and IdU fibers lengths were measured and plotted for the indicated genotypes. The fibers were evaluated for continuing forks (CldU followed by IdU), stalled forks (CldU alone), or new forks (IdU alone) and graphed as percentage of all fibers evaluated. Controls are represented by: *Brca2*^*+/+*^, *Brca2*^*Ko/+*^.
(PDF)

S1 Table List of primers. (DOCX)
