# Supplementary figures and images for: Interaction with PALB2 Is Essential for Maintenance of Genomic Integrity by BRCA2
Source: PLoS Genet. 2016 Aug 4;12(8):e1006236. doi: 10.1371/journal.pgen.1006236 (PMC4973925; doi:10.1371/journal.pgen.1006236)

# S1 Fig

**A**

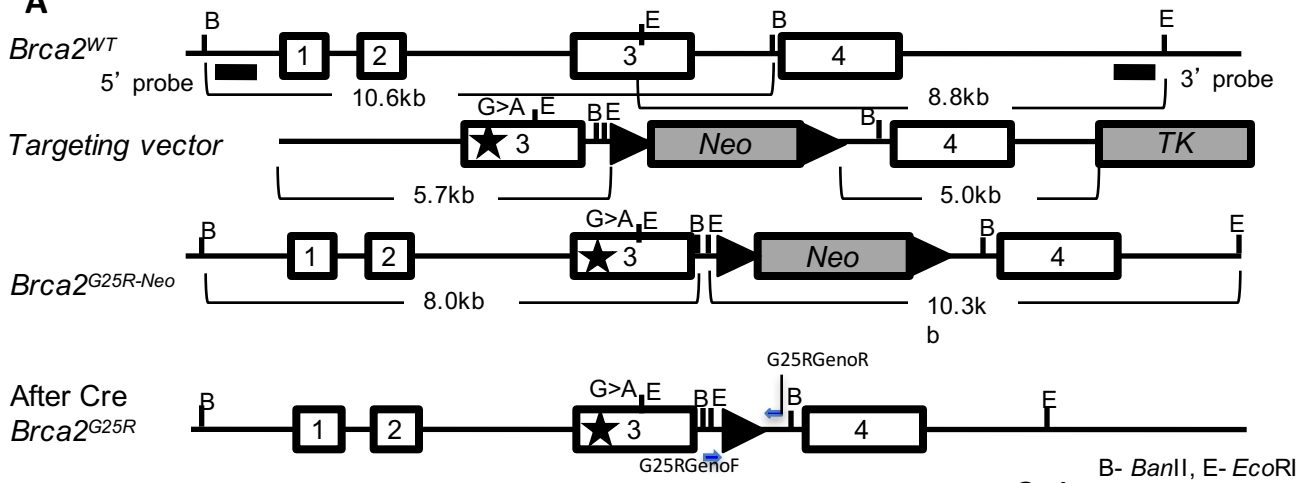

**B**

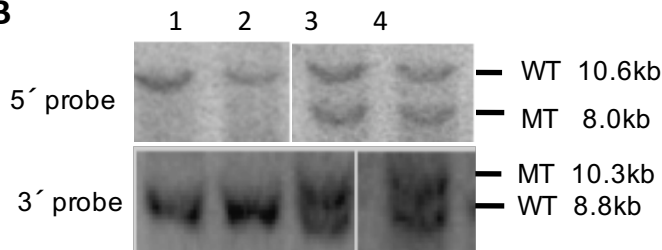

**C**

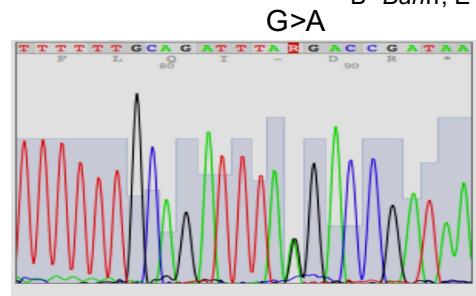

**D**

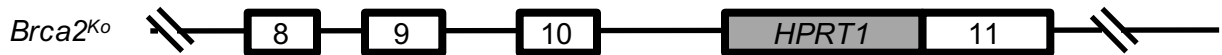

**E**

*Brca2*<sup>G25R</sup> Genotyping PCR

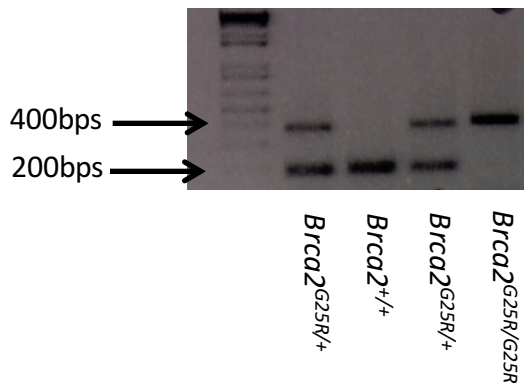

Supplement: S1 Fig — A: Schematic representation of the gene targeting strategy to generate the Brca2G25R knock-in allele showing the wild type locus (Brca2WT), targeting vector, targeted allele (Brca2G25R-Neo) showing the presence of loxP-Neo-loxP cassette and the Brca2G25R knock-in allele with the point mutation and a single loxP site. The first four exons of Brca2 exons are indicated as boxes with corresponding numbers. Location of restriction sites used for Southern-based genotyping are indicated by B (BanII) and E (EcoRI). G25RGenoF and G25RGenoR designate location and direction of primers (in blue) used for PCR-based genotyping. Asterisk marks the location of the G>A mutation in the first base of codon 25 of Brca2 in exon 3. Location of probes used to detect 5′ and 3′ end targeting are shown with a solid box below the Brca2WT locus. B. Southern blot analysis showing correct gene targeting. D. Sequence read confirming G>A substitution in codon 25 of Brca2. D. Schematic representation of the Brca2 null allele (Brca2Ko/+) showing deletion of a portion of exon 11 which is replaced with human HPRT1 minigene. E. PCR-based genotyping of Brca2G25R allele utilizing the primers show in blue arrows in A. (PDF) [file pgen.1006236.s001.pdf]

S2 Fig

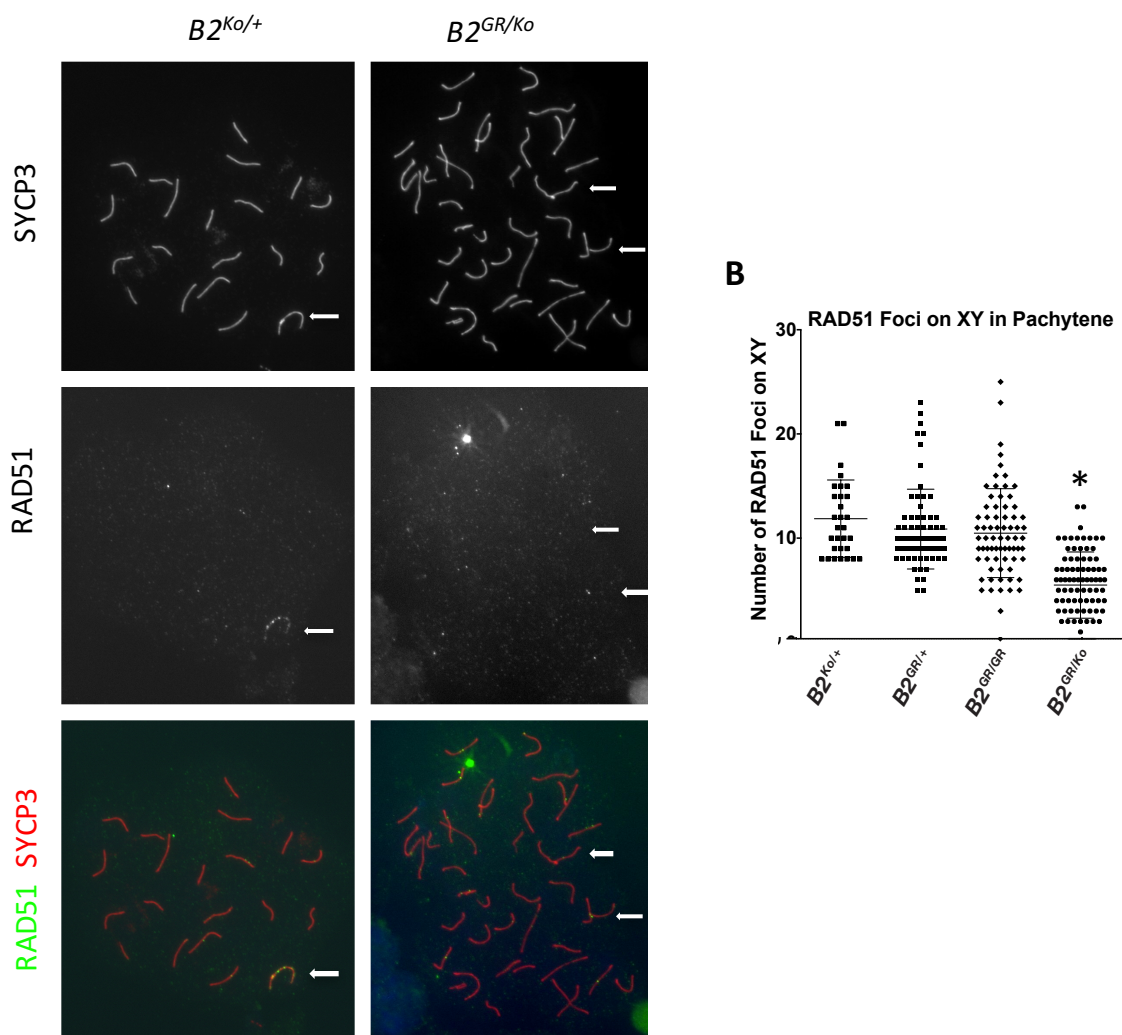

Supplement: S2 Fig — A. Representative meiocytes at pachynema of (B2Ko/+, left and B2GR/Ko, right) stained with SYCP3 (top, red), and RAD51 (middle, green) and merged (Bottom), arrows indicate the XY Body. B. Quantification of RAD51 foci along the XY body. Error bars: SD, * p<0.05. Controls are represented by either: Brca2Ko/+ or Brca2G25R/+. Abbreviations: Brca2Ko/+ = B2Ko/+, Brca2G25R/+ = B2GR/+, Brca2G25R/G25R = B2GR/GR, Brca2G25R/Ko = B2GR/Ko. (PDF) [file pgen.1006236.s002.pdf]

S3 Fig

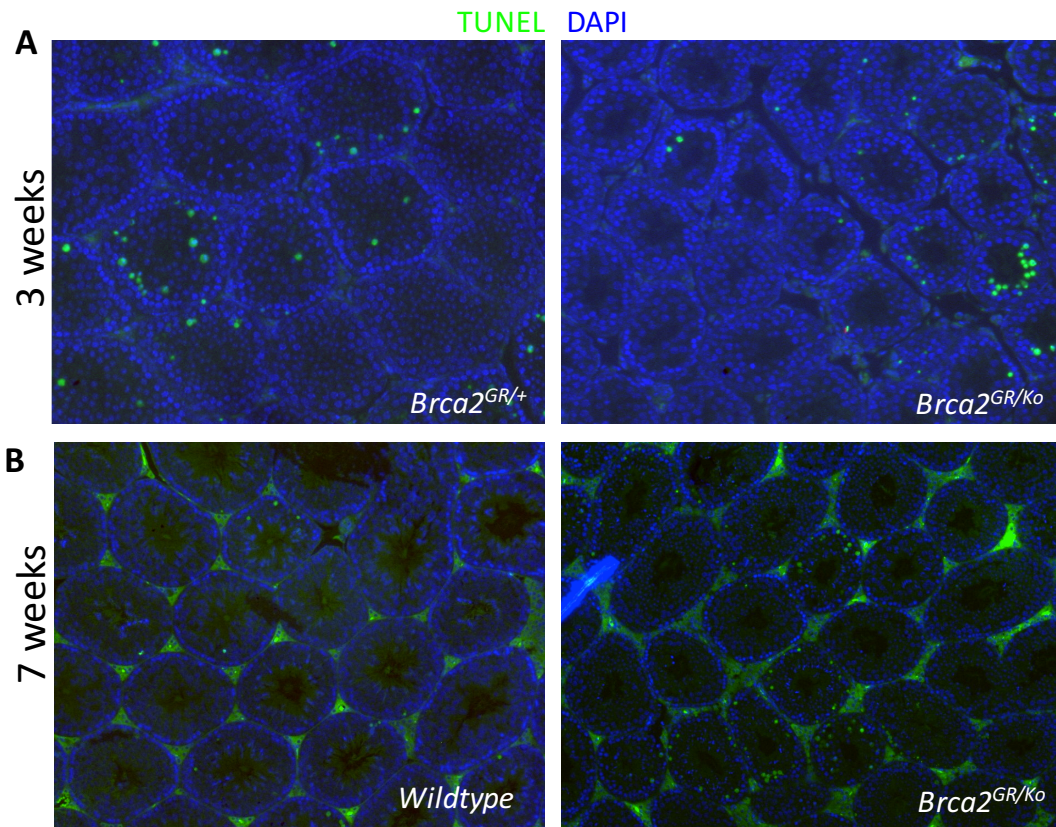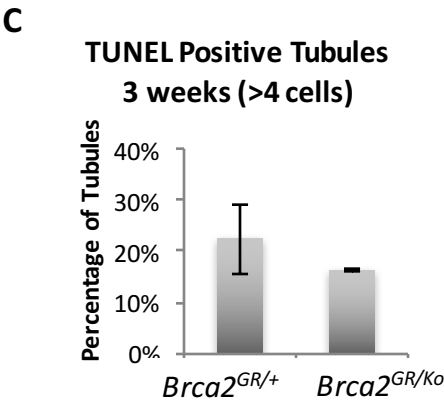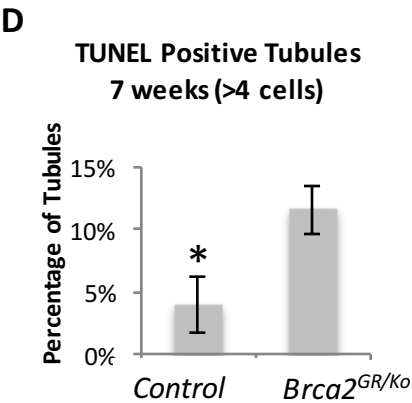

Supplement: S3 Fig — Representative images of testes with TUNEL staining (green) and counterstained with DAPI (blue) to show the cells that are undergoing apoptosis at 3 weeks (A.) and 7 weeks (B.). Quantification of TUNEL positive cells at 3 weeks (C.) and 7 weeks (D.). Error Bars: SD, *p<0.05. (PDF) [file pgen.1006236.s003.pdf]

S4 Fig

A

Female

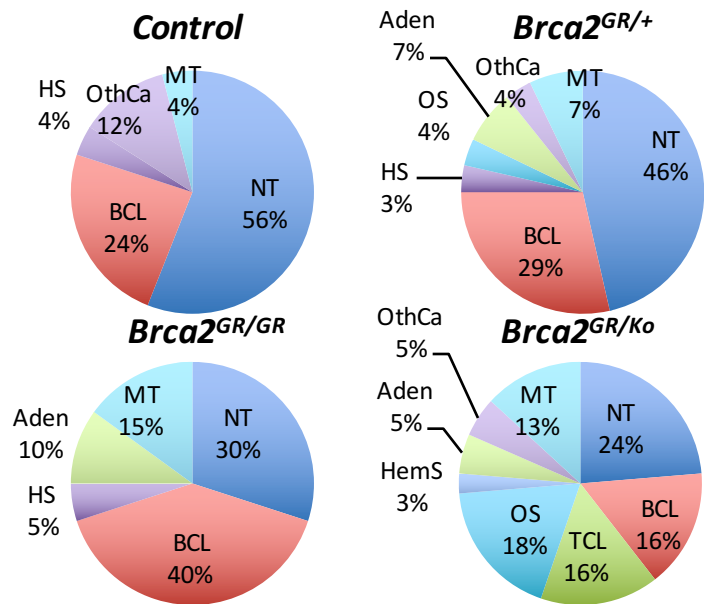

B

Male

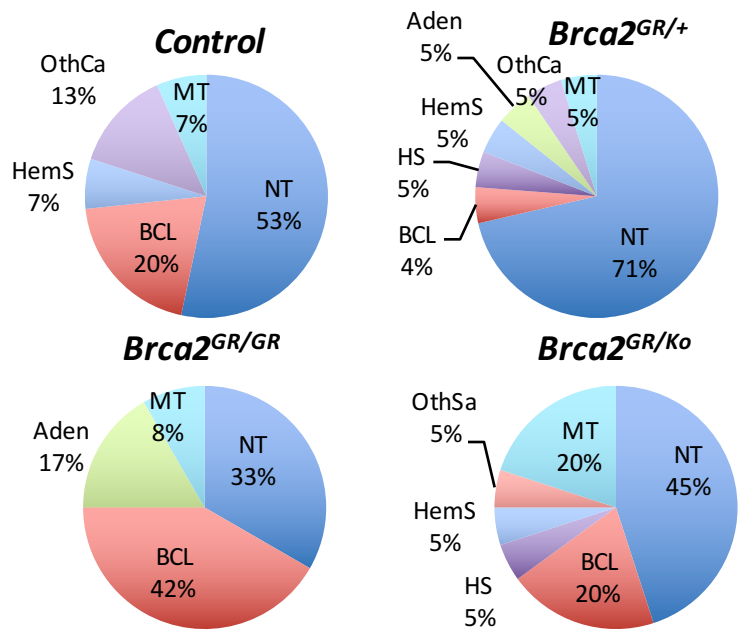

Supplement: S4 Fig — Distribution of tumor types found in mice by gender of indicated genotypes. A. Females and B. Males. Abbreviations: NT = No tumor observed, BCL = B-cell lymphoma, TCL = T-cell lymphoma, HS = Histiocytic sarcoma, OS = Osteosarcoma, HemS = Hemangiosarcoma, OthSa = Other sarcomas, AD = Adenomas, OthCa = Other carcinomas, MT = Multiple tumors. (PDF) [file pgen.1006236.s004.pdf]

S5 Fig

A

Female *Trp53*<sup>Ko/+</sup>

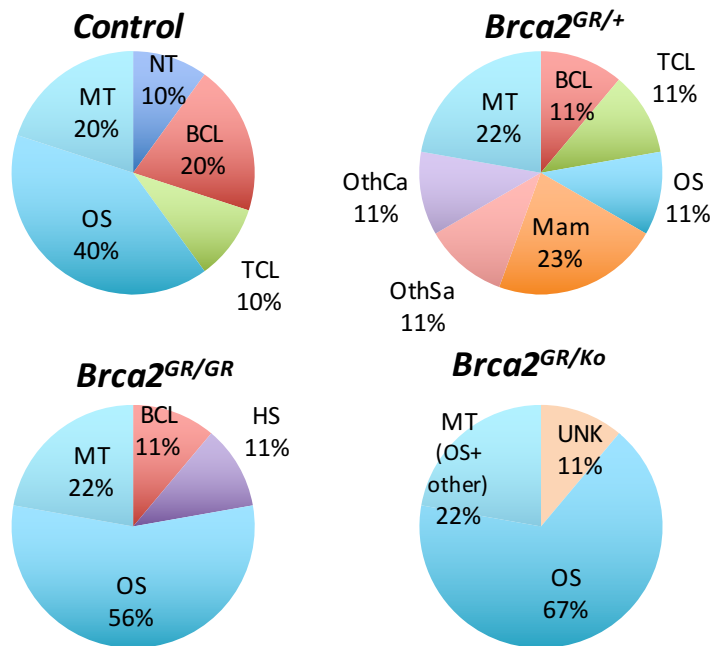

B

Male *Trp53*<sup>Ko/+</sup>

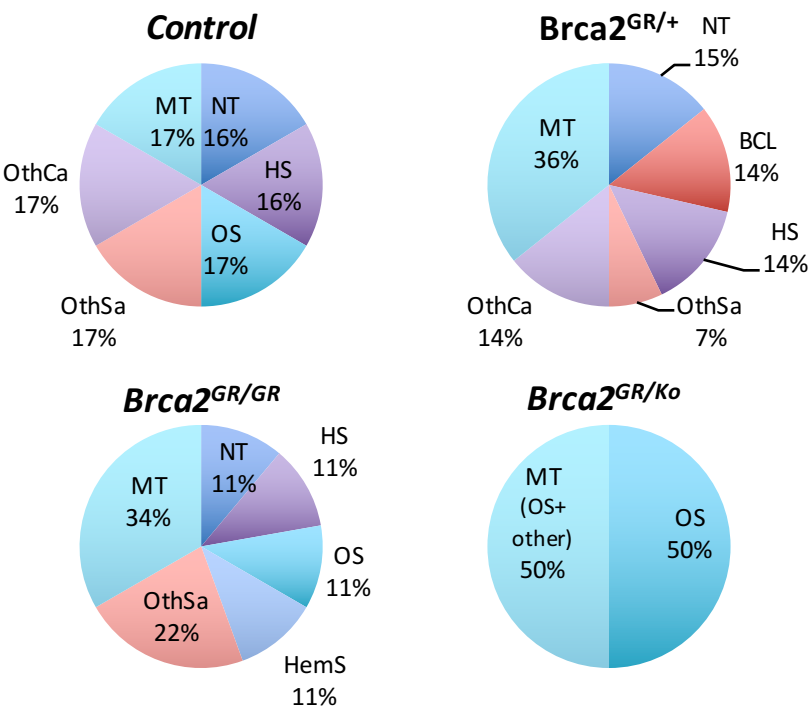

Supplement: S5 Fig — Distribution of tumor types found in mice of indicated genotypes. A. Females and B. Males. Abbreviations: NT = No tumor observed, BCL = B-cell lymphoma, TCL = T-cell lymphoma, HS = Histiocytic sarcoma, OS = Osteosarcoma, HemS = Hemangiosarcoma, OthSa = Other sarcomas, AD = Adenomas, OthCa = Other carcinomas, MT = Multiple tumors, UNK = cause of death unknown. (PDF) [file pgen.1006236.s005.pdf]

S6 Fig

A

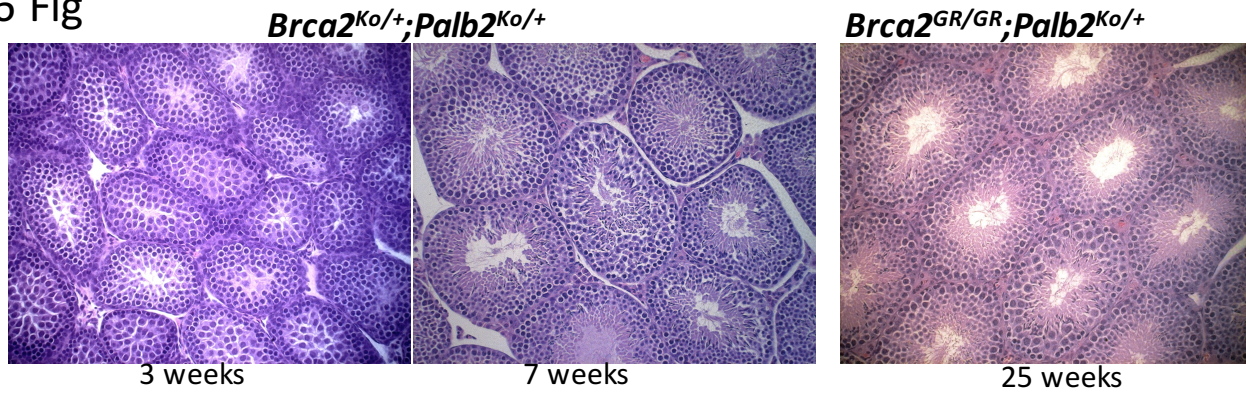

B

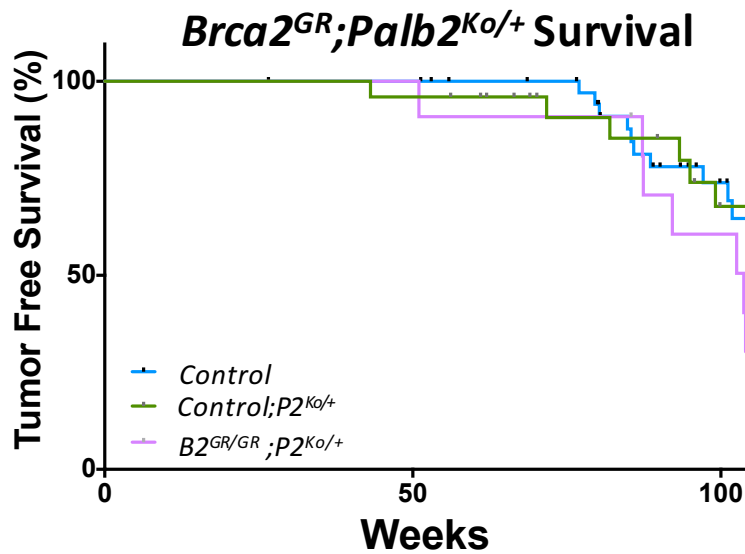

C

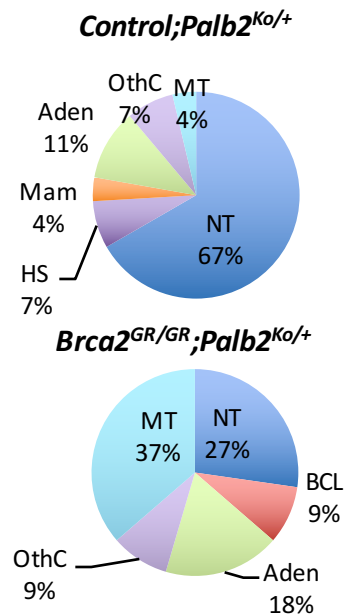

D

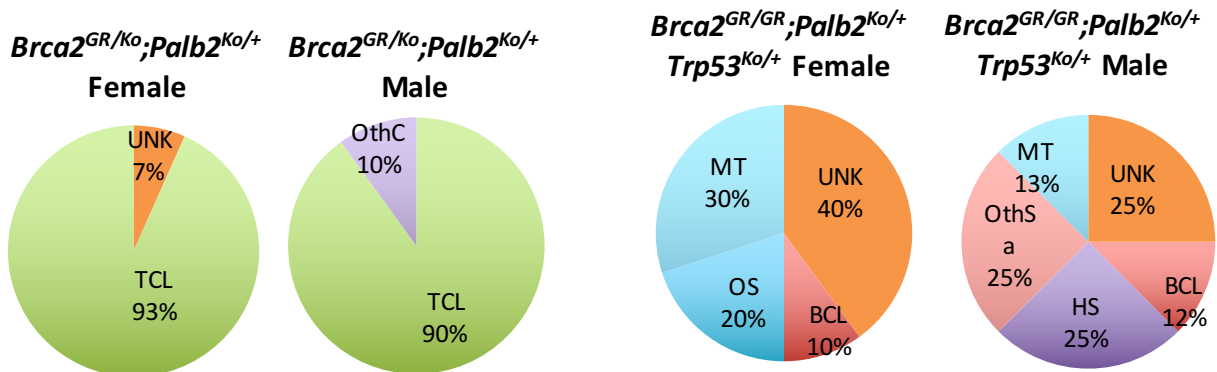

Supplement: S6 Fig — A. Testis cross section of Brca2Ko/+;Palb2Ko/+ mice showing normal spermatogenesis at 3 weeks and 7 weeks of age. Far left panel: Testis cross section of Brca2GR/GR;Palb2Ko/+ mouse at 25 weeks showing normal spermatogenesis. B. Kaplan-Meier tumor-free survival curves of Control, Control;Palb2Ko/+ and Brca2GR/GR;Palb2Ko/+ mutant animals (104 week study). C. Total distribution of tumors found in indicated genotypes. D. Tumor distribution broken down by gender of indicated genotypes. Abbreviations: NT = No tumor observed, BCL = B-cell lymphoma, TCL = T-cell lymphoma, HS = Histiocytic sarcoma, OS = Osteosarcoma, HemS = Hemangiosarcoma, OthSa = Other sarcomas, AD = Adenomas, OthCa = Other carcinomas, MT = Multiple tumors, UNK = cause of death unknown. Controls are represented by: Brca2+/+ and Brca2Ko/+. Abbreviations: Brca2G25R/G25R = B2GR/+, Palb2Ko/+ = P2Ko/+. (PDF) [file pgen.1006236.s006.pdf]

S8 Fig

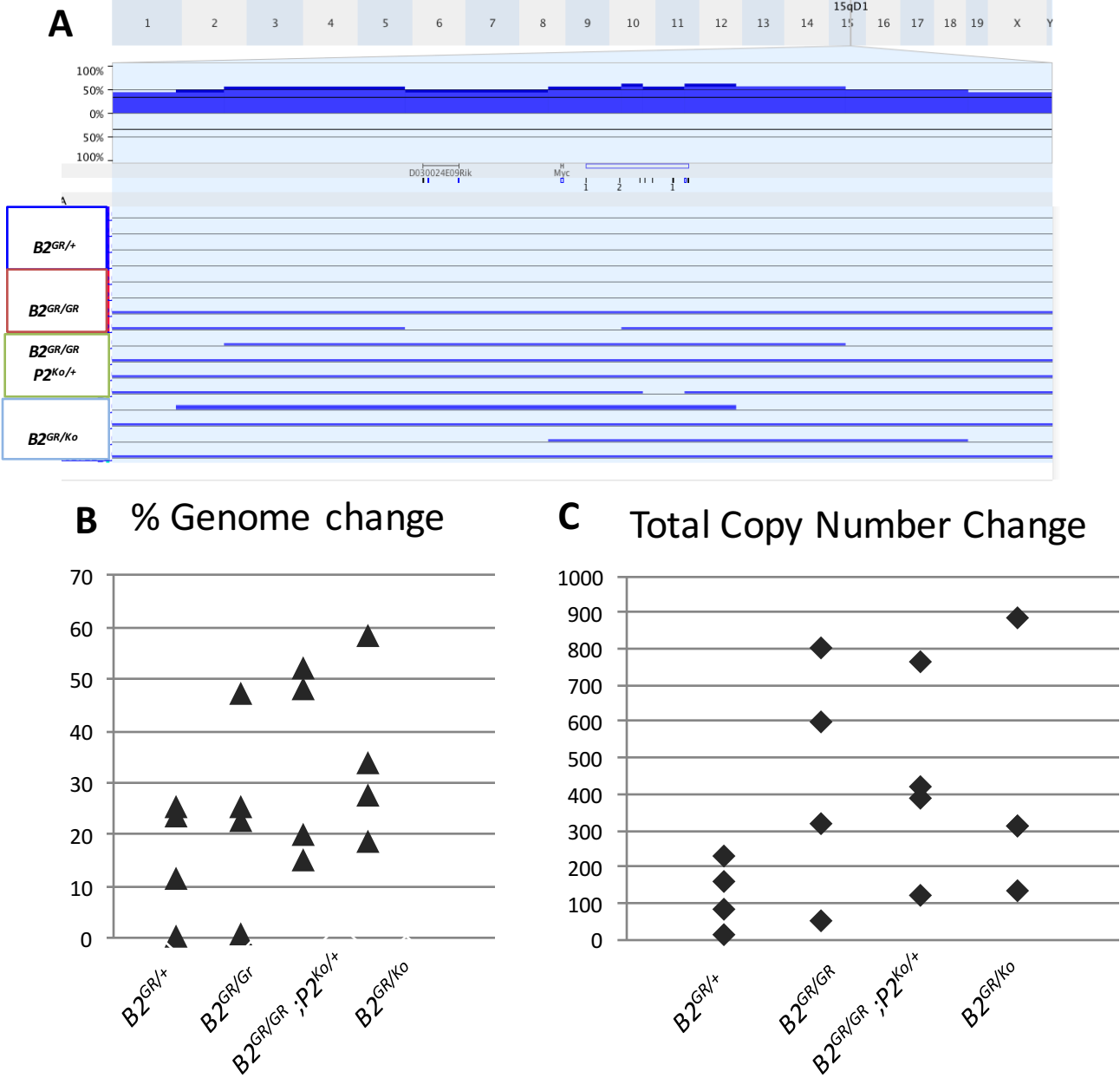

Supplement: S8 Fig — A. Image of amplified region around the myc locus in Brca2G25R/G25R Palb2Ko/+ and Brca2G25R/Ko tumors on a Trp53Ko/+ genetic background. B. Percent genome change, and C. total number of copy number changes in the individual tumors. Controls are represented by: Brca2G25R/+; Trp53Ko/+. Abbreviations: Brca2G25R/+ = B2GR/+, Brca2G25R/G25R = B2GR/GR, Brca2G25R/Ko = B2GR/Ko, Palb2Ko/+ = P2Ko/+, Trp53Ko/+ = p53Ko/+. (PDF) [file pgen.1006236.s008.pdf]

A

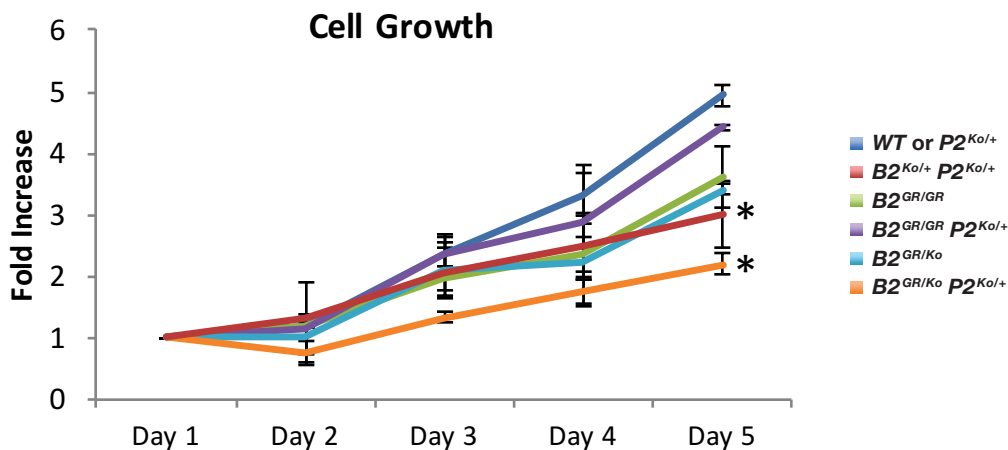

B

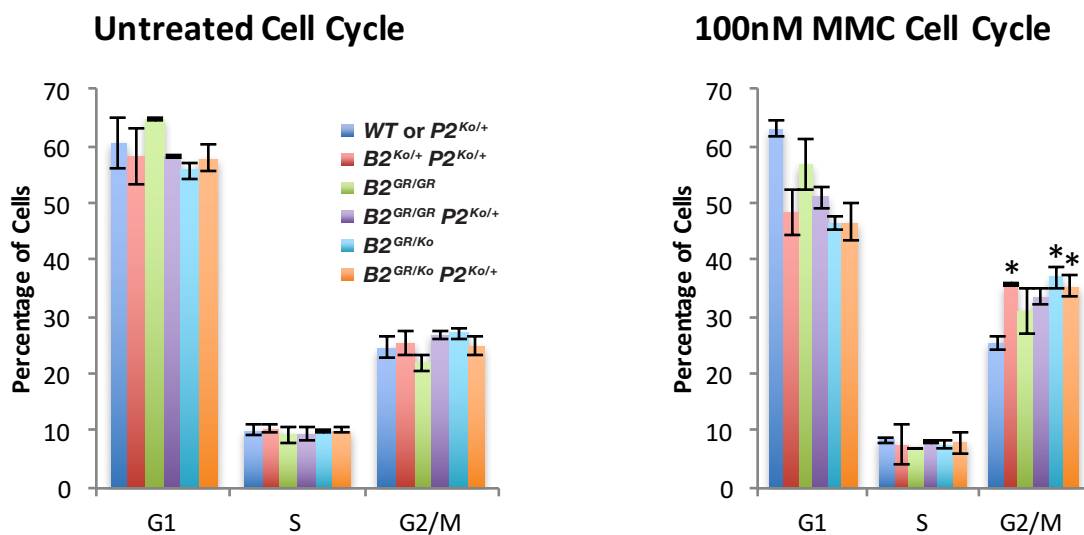

C

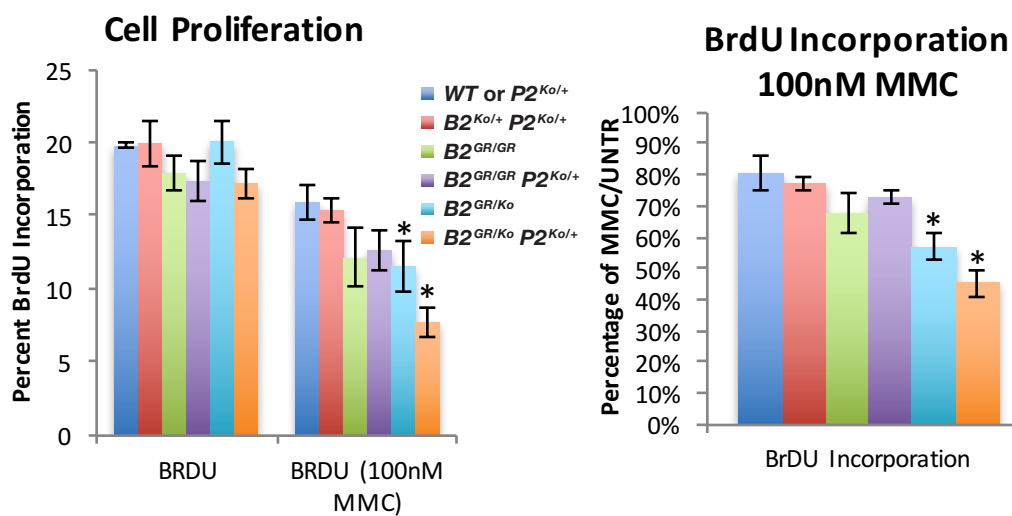

Supplement: S9 Fig — A. Fold change of cell growth over the course of 5 days of MEFs of various genotypes. B. Untreated and 100nM MMC treated cell cycle profiles of MEFs of the various genotypes. C. BrdU incorporation of untreated and 100nM MMC treated MEFs of the various genotypes. Error Bars: SD, *p<0.05. Controls are represented by: Brca2+/+ (with or without Palb2Ko/+). Abbreviations: Brca2+/+ = WT, Brca2Ko/+ = B2Ko/+, Brca2G25R/G25R = B2GR/GR, Brca2G25R/Ko = B2GR/Ko, Palb2Ko/+ = P2Ko/+. (PDF) [file pgen.1006236.s009.pdf]

# S10 Fig

**A**

## RAD51 Foci in PCNA positive cells

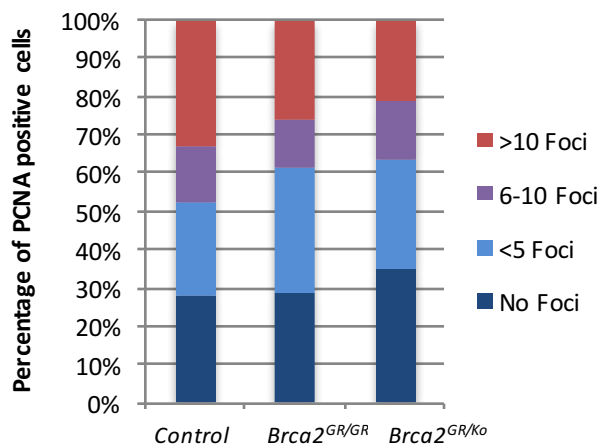

**B**

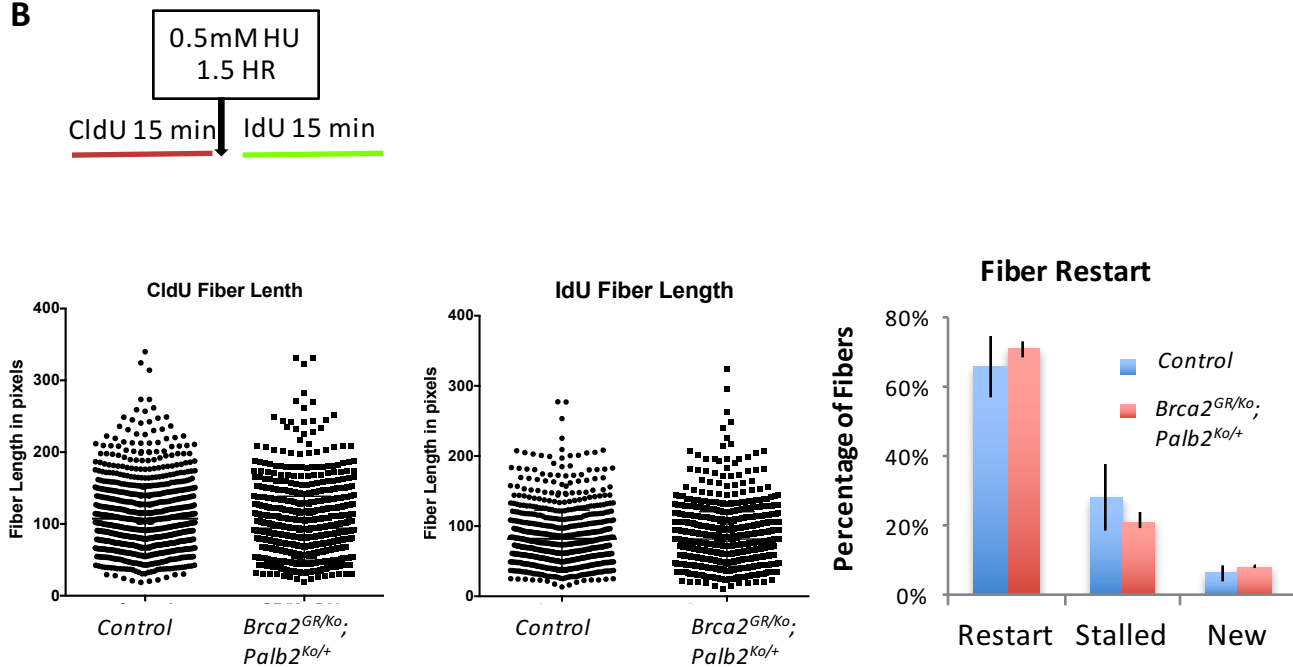

Supplement: S10 Fig — A. RAD51 foci quantification in cells that are PCNA positive in indicated genotypes. B. Fork restart assay: The cells were pulsed with CldU for 15 minutes, then 0.5mM HU was added for 1.5 hrs to stall replication and then released into media containing IdU for 15 minutes. CldU and IdU fibers lengths were measured and plotted for the indicated genotypes. The fibers were evaluated for continuing forks (CldU followed by IdU), stalled forks (CldU alone), or new forks (IdU alone) and graphed as percentage of all fibers evaluated. Controls are represented by: Brca2+/+, Brca2Ko/+. (PDF) [file pgen.1006236.s010.pdf]
