# Supplementary material for: Interaction with PALB2 Is Essential for Maintenance of Genomic Integrity by BRCA2
Source: PLoS Genet. 2016 Aug 4;12(8):e1006236. doi: 10.1371/journal.pgen.1006236 (PMC4973925; doi:10.1371/journal.pgen.1006236)

# S7 Fig

*Brca2*$^{GR/+}$;*Trp53*$^{Ko/+}$

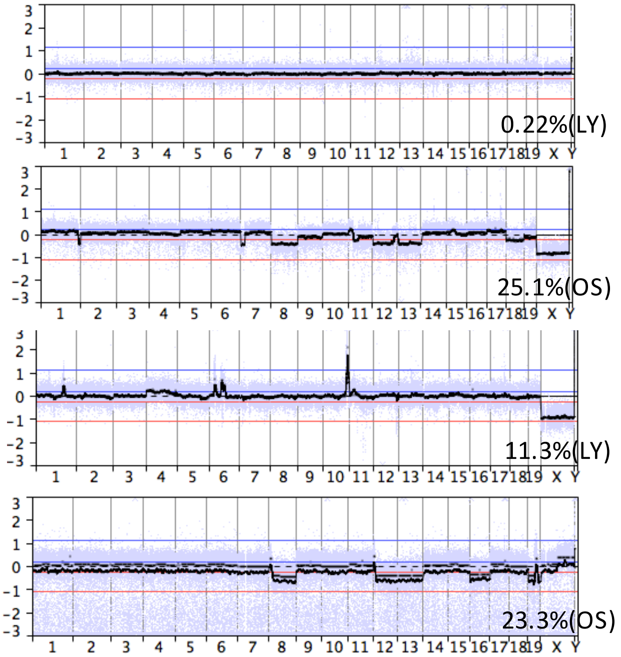


*Brca2*$^{GR/GR}$;*Trp53*$^{Ko/+}$

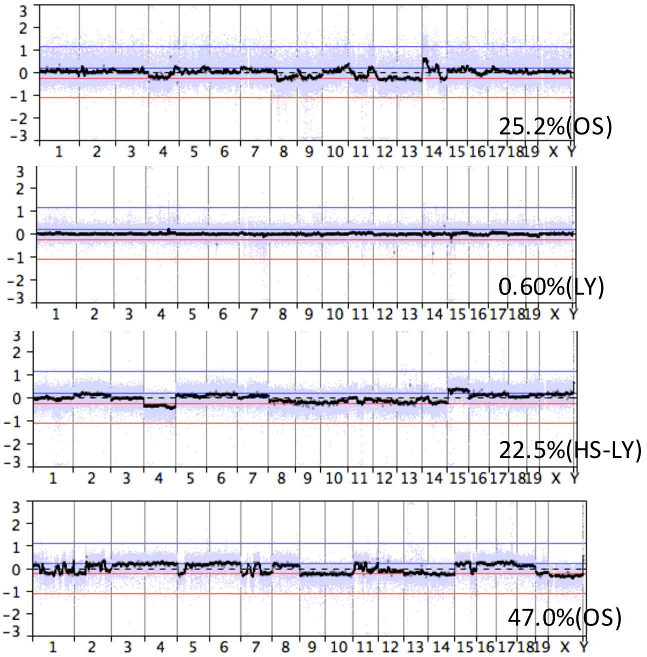


*Brca2*$^{GR/Ko}$;*Trp53*$^{Ko/+}$

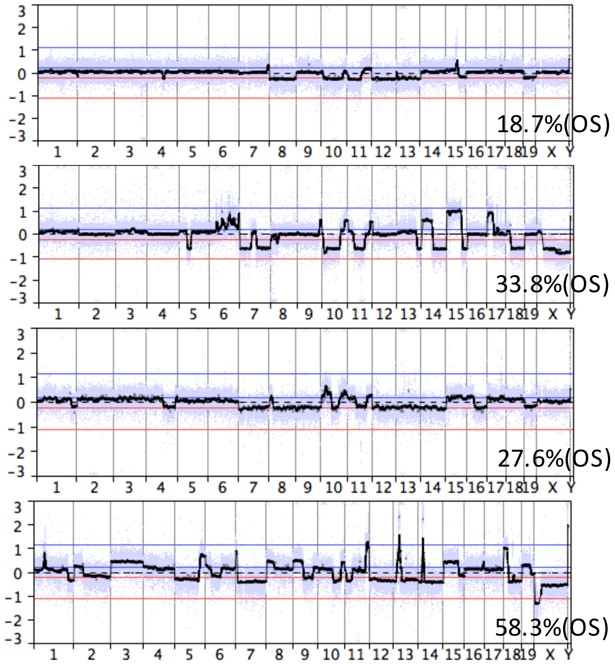


*Brca2*$^{GR/GR}$;*Palb2*$^{Ko/+}$;*Trp53*$^{Ko/+}$

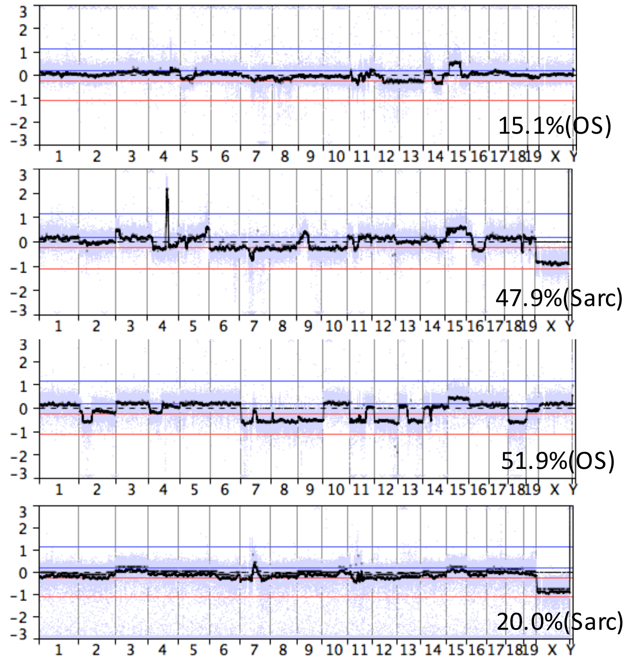

Supplement: S7 Fig — Individual copy number variation (CNV) graphs for each of the heterogeneous tumors across the genome. Chromosome number is at the bottom and amount of gain of loss is indicated at the Y-axis. (+1 or -1 indicates a 2 copy gain or 2 copy loss) Abbreviations: Ly = Lymphoma, OS = Osteosarcoma, HS = Histiocytic Sarcoma, Sarc = Sarcoma. (PDF) [file pgen.1006236.s007.pdf]
