# Supplementary material for: Interaction with PALB2 Is Essential for Maintenance of Genomic Integrity by BRCA2
Source: PLoS Genet. 2016 Aug 4;12(8):e1006236. doi: 10.1371/journal.pgen.1006236 (PMC4973925; doi:10.1371/journal.pgen.1006236)
Supplement: S1 Table — (DOCX) [file pgen.1006236.s011.docx]

**S1 Table: List of Primers**

*Brca2^G25R^* Genotyping:

G25RgenoF: GCCTCTTCTTTCTTCCTATGCTT

G25RgenoR: CACCTGGCACATAGGTCAGAG

Wildtype- 193 bps

Mutant ~ 400bps

*Brca2^Ko/+^* Genotyping:

HPRT3: aagtgttggatataagccag

SKS301-R: cccactagctgtatgaaaac

SKS302-F: gcaaaagtaggaccaagagg

Wildtype -816bps

Mutant ~1000bps

*Palb2^Ko/+^* Genotyping:

Palb2gtF: gagcaccagaggagatcc

Palb2wtF: ccagcagaaaagaaggacc

Palb2wtR: gttcccttagcagaagtgc

Wildtype-361bps

Mutant~500bps
